# Hexosamine Pathway Disruption by GFPT1 Loss Drives Coordinated Defects in Glycosylation, Autophagy, and Trafficking

**DOI:** 10.3390/biom16070966

**Published:** 2026-06-30

**Authors:** Stephen H. Holland, Ricardo Carmona-Martinez, Andreas Hentschel, Alexa Derksen, Kaela O’Connor, Daniel O’Neil, Kelly Ho, Stephen D. Baird, Andreas Roos, Sally Spendiff, Hanns Lochmüller

**Affiliations:** 1 Children’s Hospital of Eastern Ontario Research Institute, Ottawa, ON K1H 8L1, Canada; 2 Department of Cellular and Molecular Medicine, Faculty of Medicine, University of Ottawa, Ottawa, ON K1H 8M5, Canada; 3 Dr. Eric Poulin Center for Neuromuscular Disorders, Brain and Mind Research Institute, University of Ottawa, Ottawa, ON K1H 8M5, Canada; 4 Leibniz-Institute for Analytical Sciences–ISAS-e.V, 44139 Dortmund, Germany; 5 Department of Pediatric Neurology, Centre for Neuromuscular Disorders in Children, University Duisburg-Essen, Hufelandstrasse 55, 45122 Essen, Germany; 6 Department of Neurology with Heimer Institute for Muscle Research, University Hospital Bergmannsheil, 44789 Bochum, Germany; 7 Division of Neurology, Department of Medicine, The Ottawa Hospital, Ottawa, ON K1H 8L6, Canada; 8 Department of Neuropediatric and Muscle Disorders, Medical Center, Faculty of Medicine, University of Freiburg, 79106 Freiburg, Germany; 9 Centro Nacional de Analisis Genomico (CNAG-CRG), Center for Genomic Regulation, Barcelona Institute of Science and Technology (BIST), 08028 Barcelona, Spain

**Keywords:** congenital myasthenic syndrome, GFPT1, trafficking, autophagy, glycosylation

## Abstract

Glutamine-Fructose-6-Phosphate Transaminase 1 (GFPT1), the rate-limiting enzyme of the hexosamine biosynthetic pathway (HBP), provides the UDP-N-acetylglucosamine (UDP-GlcNAc) required for protein glycosylation. Biallelic mutations in *GFPT1* cause congenital myasthenic syndromes (*GFPT1*-CMS), yet the molecular mechanisms linking impaired glycosylation to skeletal muscle dysfunction remain incompletely understood. Here, we combine cellular models of inducible *Gfpt1* knockdown and a skeletal muscle-specific *Gfpt1* knockout mouse (*Gfpt1^Tm1d/Tm1d^*) with whole-cell proteomics, immunoblot studies and secretomics to define glycosylation-dependent defects in intracellular trafficking, ER stress signaling and autophagy. Global proteomic profiling of *Gfpt1*-deficient myoblasts revealed marked downregulation of protein trafficking pathways and impaired secretion of key muscle cargo proteins, including serglycin (Srgn). Loss of GFPT1 reduced both high-molecular-weight glycosylated serglycin and its core protein, accompanied by intracellular retention and decreased secretion. These trafficking defects coincide with robust activation of the unfolded protein response (UPR), evidenced by increased *Xbp1* expression and accumulation of spliced Xbp1s across pharmacologic, cellular, and mouse models of *GFPT1* deficiency. Converging evidence from proteomics, immunoblotting, and immunofluorescence demonstrated impaired autophagy, including increased LC3-II accumulation, elevated p62/Sqstm1 levels, and enhanced p62-positive puncta in both *Gfpt1*-deficient C2C12 myoblasts and skeletal muscle. Soluble/insoluble fractionation further confirmed p62 accumulation, indicating defective autophagic flux and buildup of aggregated cargo. Together, these findings identify a glycosylation-dependent failure in protein trafficking that triggers ER stress, UPR activation, and autophagy impairment in *Gfpt1*-deficient skeletal muscle. This mechanistic cascade provides a unifying explanation for muscle pathology in *GFPT1*-CMS and suggests that restoring glycosylation or improving proteostasis may represent viable therapeutic approaches.

## 1. Introduction

Protein glycosylation is essential for proper folding, stability, secretion and intracellular trafficking of thousands of proteins across mammalian cell types [1,2,3]. Glycoproteins represent nearly half of the mammalian proteome, and defects in glycosylation disrupt critical processes—particularly in highly secretory or structurally demanding tissues such as skeletal muscle [3]. The hexosamine biosynthetic pathway (HBP) provides the essential nucleotide sugars that fuel these glycosylation reactions, with UDP-GlcNAc serving as a central substrate for N-, O-linked glycosylation and O-GlcNAcylation [4,5]. At the apex of this pathway sits glutamine-fructose-6-phosphate transaminase 1 (GFPT1), the rate-limiting enzyme that controls the metabolic flux into the hexosamine biosynthetic pathway (HBP) by converting fructose-6-phosphate into glucosamine-6-phosphate [4,5]. Since GFPT1 function determines the availability of UDP-GlcNAc, it acts as a critical metabolic gatekeeper linking nutrient status, cellular stress responses, and post-translational protein modifications [4,5,6]. Even modest reductions in HBP flux can compromise protein folding, vesicle export, and quality control systems that depend on glycan maturation, underscoring the central role of GFPT1 in maintaining cellular proteostasis.

Congenital myasthenic syndromes (CMSs) are a group of inheritable, early-onset neuromuscular disorders caused by mutations in proteins critical for the development, maintenance and function of the neuromuscular junction (NMJ) [7,8]. Clinically, CMS is characterized by fatigable muscle weakness and exhibits considerable genetic and phenotypic heterogeneity [7,8,9]. To date, approximately 40 genes have been implicated in CMS, with a subset of five genes linked to defects in protein glycosylation [8,10,11,12,13]. Biallelic pathogenic variants to *GFPT1* lead to reduced protein abundance and impaired enzymatic activity [10,14,15,16,17,18,19,20,21,22]. Patients with *GFPT1*-CMS typically present with predominant limb-girdle muscle weakness [10,14,15]. Histopathological analysis of patient muscle biopsies reveals diverse myopathic features, including tubular aggregates and rimmed vacuoles [15,16]. Notably, these pathological hallmarks are recapitulated in our skeletal muscle-specific *Gfpt1* knockout mouse model (*Gfpt1^tm1d/tm1d^*), validating its relevance to human *GFPT1*-CMS [6,22,23]. Recent work from our group demonstrated that *Gfpt1* depletion disrupts multiple glycosylation processes, including protein O-GlcNAcylation and N-linked glycosylation [6,23]. Although *GFPT1*-deficiency is known to impact protein glycosylation, the downstream mechanisms linking disrupted glycosylation to skeletal muscle pathology remain poorly understood.

Protein glycosylation is intimately coupled to intracellular trafficking and endoplasmic reticulum (ER) homeostasis. Hypoglycosylation disrupts protein folding in the ER, leading to protein misfolding, ER retention, and preferential targeting to ER-associated degradation (ERAD) due to failure to pass glycan-dependent quality control checkpoints [24,25]. When misfolded proteins accumulate, cells activate the unfolded protein response (UPR) to restore proteostasis by enhancing chaperone expression, attenuating translation, and upregulating ERAD [26]. In parallel, disruptions in protein folding and trafficking can impair autophagy, a degradative pathway essential for the clearance of damaged proteins, aggregated cargo, and dysfunctional organelles [27,28,29]. A hallmark of impaired autophagic flux is the accumulation of p62 and LC3-II, reflecting defective cargo degradation and contributing to the formation of protein aggregates [30]. Intriguingly, prior studies with *Gfpt1^tm1d/tm1d^* mice demonstrated a ~2.5-fold increase in p62, suggesting that autophagy may be impaired within *Gfpt1*-deficient models [22].

Here, we combine whole-cell proteomics, secretomics, cellular models of *Gfpt1*-depletion and a *Gfpt1^tm1d/tm1d^* mouse model to define how Gfpt1 deficiency disrupts proteostasis within skeletal muscle. We identify downregulation of vesicle trafficking proteins, intracellular retention of secreted proteins such as serglycin (Srgn), activation of the UPR pathway and a profound impairment of autophagic flux characterized by p62 and LC3-II accumulation. Together, our findings reveal a glycosylation-dependent cascade in which defective trafficking and ER stress converge on impaired autophagy, providing a unified mechanistic framework for muscle pathology in *GFPT1*-CMS.

## 2. Methods

### 2.1. Cell Culture

C2C12 cells (ATCC, CRL-1722, Burlington, ON, Canada) were cultured in high-glucose Dulbecco’s Modified Eagle Medium (DMEM) supplemented with 10% fetal bovine serum (FBS), 1% glutamine, and 1% penicillin/streptomycin. A *Gfpt1*-deficient C2C12 cell model was established using a TET-ON miR-30 tetracycline-inducible system with two different short hairpin sequences targeting *Gfpt1* (shGfpt1 #1 and shGfpt1 #2) (VectorBuilder, Chicago, IL, USA) as previously described [6]. *Gfpt1* silencing was achieved by doxycycline treatment at 2 μg/mL for 72 h (Sigma, D9891, Toronto, ON, Canada).

### 2.2. Animal Husbandry

Animals were housed at the University of Ottawa Animal Care and Veterinary Facility (Roger Guidon Hall, Ottawa, ON, Canada), and all breeding and experimental protocols were approved by the internal Animal Care Committee (protocols: breeding—CHEO-3089 and experimental—CHEO-3120 approved on 16 November 2018 and 2 April 2019, respectively). Mice were maintained on a 12 h light–dark cycle, with access to food and water ad libitum for the duration of this study. Mice were generated and genotyped as previously reported [6,22,23]. For this study, Tm1C homozygous mice (which contain two LoxP sites flanking the seventh exon of *Gfpt1* but lack *Ckm-Cre* expression) were used as controls. *Gfpt1^tm1d/tm1d^* mice contain two LoxP sites flanking the 7th exon of *Gfpt1* but also express a Cre recombinase tied to the *Ckm* reporter, which creates a skeletal muscle-specific knockout of *Gfpt1* [6,22].

### 2.3. Sample Preparation and Protein Digestion

*Gfpt1*-deficient and scramble control cells were seeded into separate 15 cm plates. At 75% confluency, *Gfpt1* silencing was achieved by doxycycline treatment at 2 μg/mL for 72 h (Sigma, D9891). Prior to collection, cells were serum starved for three hours, followed by a replacement with growth media for one hour, and finally serum starved for an additional 4 h. Afterwards, conditioned media were collected, centrifuged at 1000× *g* for 15 min. The supernatant was collected and snap frozen. This sample represents the secretome.

Frozen cell pellets were treated with 200 µL of lysis buffer (50 mM TEAB, pH 7.8, 5% SDS, supplemented with cOmplete™ ULTRA protease inhibitor cocktail (Roche, #0589297001, Penzberg, Germany) followed by mechanical disruption using a Bioruptor^®^ sonication system (Diagenode, Seraing, Belgium) for 10 min (30 s on/30 s off, 10 cycles) at 4 °C. To achieve complete homogenization, samples were additionally subjected to probe sonication (30 s, 1 s on/1 s off, amplitude 40%), and insoluble material was removed by centrifugation (20,000× *g*, 15 min, 4 °C). Protein concentration in the cleared supernatant was quantified using DC Protein Assay (BioRad, 5000111, Dreieich, Germnay) with BSA dilutions (Pierce, 23209, Dreieich, Germnay) as standards according to the manufacturer’s instructions.

Reduction of disulfide bonds was performed by incubation with 10 mM TCEP at 37 °C for 30 min, followed by alkylation with 15 mM IAA at room temperature for 30 min in the dark. For proteolytic digestion, 100 µg of total protein from each sample was processed using the S-Trap protocol (Protifi, New York, NY, USA) with a protein-to-trypsin ratio of 20:1. Digestion was carried out for 2 h at 42 °C, and the reaction was terminated by acidification with formic acid to a final pH below 3.

The completeness of proteolytic cleavage was evaluated following desalting by monolithic column separation (PepSwift PS-DVB PL-CAP200-PM, Dionex) using an UltiMate 3000 HPLC system (Dionex, Germering, Germany). For each analysis, 1 µg of digest was injected directly. Chromatographic separation was achieved with a binary gradient (solvent A: 0.1% TFA; solvent B: 0.08% TFA in 84% acetonitrile), starting at 5–12% B over 5 min, followed by 12–50% B over 15 min, at a flow rate of 2.2 µL/min and 60 °C. UV detection was carried out at 214 nm [31].

### 2.4. DDA-LC-MS/MS Analysis

A total of 1 µg of the prepared samples was analyzed using an UltiMate 3000 RSLC nano UHPLC system paired with a QExactive HF mass spectrometer (Both ThermoFisher Scientific, Breman, Germnay). Initially, the samples were loaded onto a 75 µm × 2 cm, 100 Å, C18 pre-column at a flow rate of 20 µL/min for 20 min. Subsequently, separation was carried out on a 75 µm × 50 cm, 100 Å, C18 main column with a flow rate of 250 nL/min, utilizing a binary gradient (solvent A: 0.1% formic acid (Sigma-Aldrich, Hamburg, Germany; solvent B: 84% acetonitrile (Sigma-Aldrich, Hamburg, Germany)) with 0.1% formic acid; 5% B for 3 min, linear increase to 25% for 102 min, a further linear increase to 33% for 10 min, and a final linear increase to 95% for 2 min followed by a linear decrease to 5% for 5 min). For MS survey scans, the parameters included operating MS in data-dependent acquisition mode (DDA) with full MS scans from 300 to 1600 *m*/*z* (resolution 60,000) and the polysiloxane ion at 371.10124 *m*/*z* as a lock mass. The maximum injection time was set to 120 ms, and the automatic gain control (AGC) was set to 1E6. Fragmentation involved selecting the 15 most intense ions (above the threshold ion count of 5E3) at a normalized collision energy (nCE) of 27% in each cycle, following each survey scan. Fragment ions were acquired (resolution 15,000) with an AGC of 5E4 and a maximum injection time of 50 ms. Dynamic exclusion was set to 15 s.

### 2.5. DDA Data Analysis of C2C12 Cells

All MS raw data underwent processing using Proteome Discoverer software version 2.5.0.400 (Thermo Scientific, Bremen, Germany) and were subjected to a target/decoy mode search against a mouse Uniprot database (www.uniprot.org, accessed on 8 October 2019) utilizing the MASCOT and Sequest algorithm. The search parameters included precursor and fragment ion tolerances of 10 ppm and 0.02 Da for MS and MS/MS, respectively. Trypsin was designated as the enzyme with a maximum of 2 allowed missed cleavages. Carbamidomethylation of cysteine was set as a fixed modification, and oxidation of methionine was set as a dynamic modification. Percolator false discovery rate (strict) was established at 0.01 for both peptide and protein identification.

For reliable label-free quantification, only proteins identified with ≥2 unique peptides were considered for further analysis. Subsequently, the average normalized abundances (determined using Proteome Discoverer) were calculated for each protein and used to determine the ratio between the different conditions. Finally, a Student’s *t*-test with *p*-values was calculated in MS Excel. Only proteins with a *p*-value of ≤0.05 and an abundance ratio of ≥2 or ≤0.5 (upregulated as well as downregulated) were considered as regulated.

To further interpret the proteomic alterations, a list of significantly upregulated proteins (expression ratio ≥ 2.0) and downregulated proteins (expression ratio ≤ 0.5) was analyzed using the STRING database for functional enrichment. Gene Ontology (GO) Biological Process enrichment analysis was performed to identify overrepresented functional pathways. Protein–protein interaction networks were constructed using a minimum required interaction score of 0.7 (high confidence). Functional clustering was applied based on a similarity threshold greater than 0.7 to group proteins into related biological processes. This approach enabled the identification of key pathways and biological processes most affected by the observed proteomic changes.

### 2.6. Proteomic Analysis from Conditioned Medium

Scramble and *Gfpt1*-deficient C2C12 myoblasts were seeded into 10 cm plates. At 75% confluency, the growth medium was changed into serum-free medium for four hours, then the growth medium was applied for an hour, followed by another three-hour incubation with starvation medium. Media was collected and centrifuged to remove cell debris and snap frozen in 10 mL aliquots for proteomic analysis.

Each sample was divided into two aliquots of 1 mL cultured medium. Protein precipitation was carried out by adding 3 mL of ice-cold acetone to each aliquot, followed by incubation overnight at −20 °C. Subsequently, the samples were centrifuged at 20,000× *g* for 20 min at 4 °C to pellet the proteins. The acetone supernatant was carefully removed, and the pellets were air-dried for 3 to 5 min. To bring the protein pellets into solution, 100 μL lysis buffer (50 mM TEAB (Merck, Taufkirchen, Germany), pH 7.8; 5% SDS (ThermoFisher Scientific, Dreieich, Germnay); and cOmplete ULTRA protease inhibitor, Roche, 05892970001, Penzberg, Germany) was added to each sample. Complete lysis was ensured using a Bioruptor^®^ (Diagenode, Seraing, Belgium) for 10 min at 4 °C (30 s cycles of sonication and rest, 10 cycles total). Finally, the aliquots were combined to reconstitute the original sample. Reduction of disulfide bonds was performed by incubation with 10 mM TCEP at 37 °C for 30 min, followed by alkylation with 15 mM IAA at room temperature for 30 min in the dark. For proteolytic digestion, 100 µg of total protein from each sample was processed using the S-Trap protocol (Protifi) with a protein-to-trypsin ratio of 20:1. Digestion was carried out for 2 h at 42 °C, and the reaction was terminated by acidification with formic acid to a final pH below 3.

For the analysis of the samples acquired with nano-LC-MS/MS in DIA mode, the data were introduced to the Spectronaut software (Biognosys, Version 18.7.240506.55695) and analyzed with a direct DIA-based search. As a library, the mouse proteome data were selected from UniProt (www.uniprot.org), containing 17,175 entries (accessed on 23 August 2023). Search and extraction settings were kept as standard (BGS Factory settings). Normalization was done by the software, using global normalization based on the median. For reliable label-free quantification, only proteins identified with ≥2 unique peptides were considered for further analysis. Subsequently, the average normalized abundances (determined using Spectronaut) were calculated for each protein and used to determine the ratio between the leukocytes from the patients and the corresponding controls. Finally, a Student’s *t*-test with *p*-values was calculated in MS Excel. Only proteins with a *p*-value of ≤0.05 and an abundance ratio of ≥2 or ≤0.5 (upregulated as well as downregulated) were considered as finally regulated.

### 2.7. DIA LC-MS/MS Analysis of Conditioned Medium Samples

All samples were subjected to analysis using an UltiMate 3000 RSLC nano UHPLC system paired with a QExactive HF mass spectrometer (Both Thermo Fisher Scientific, Bremen, Germany), consistently applying 1 µg of peptide per sample. Initially, the samples were loaded onto a 75 µm × 2 cm, 100 Å, C18 pre-column at a flow rate of 20 µL/min for 20 min. Subsequently, separation was carried out on a 75 µm × 50 cm, 100 Å, C18 main column with a flow rate of 250 nL/min, utilizing a linear gradient composed of solution A (99.9% water, 0.1% formic acid) and solution B (84% acetonitrile, 15.9% water, 0.1% formic acid). The gradient length was set to 120 min (3–45% solution B). The gradient protocol included: 3% solution B for 20 min, 3–35% over 120 min, followed by three wash steps each reaching 95% solution B for 3 min. Post-wash, the instrument was allowed to equilibrate for 20 min. MS data acquisition was performed in DIA (data-independent acquisition) mode, utilizing an in-house developed spectral library. Each sample was spiked with an appropriate quantity of iRT standard (Biognosys). Full MS scans were recorded from 300 to 1100 *m*/*z* at a resolution of 60,000 (Orbitrap), using the polysiloxane ion at 445.12002 *m*/*z* as a lock mass. The automatic gain control (AGC) was set at 3 × 10^6^, with a maximum injection time of 20 milliseconds. Full MS scans were succeeded by 23 DIA windows, each covering 28 *m*/*z* with a 1 *m*/*z* overlap, starting at 400 *m*/*z*, acquired at a resolution of 30,000 (Orbitrap) with an AGC of 3E6 and a normalized collision energy (nCE) of 27 (CID).

### 2.8. Gfpt1-Deficient Myoblast Immunofluorescence Staining

1000 Scramble and *Gfpt1*-deficient C2C12 myoblasts were seeded on 96-well μ-PLATE square ibiTreat plates (Ibidi, FisherSci, 50-305-829, Toronto, ON, Canada). *Gfpt1* silencing was achieved by doxycycline treatment at 2 μg/mL for 72 h. (Sigma, D9891, Toronto, ON, Canada). Prior to staining, cells were fixed with 2% paraformaldehyde for 15 min at room temperature. Blocking buffer (5% horse serum, 1% bovine serum albumin, 0.1% Triton X-100) was incubated for 1 h. Cells were washed three times with PBS prior to primary antibody incubation overnight. Primary antibodies included: Srgn (1:100, #C-11, Santa-Cruz Biotechnology, Dallas, TX, USA) and p62 (1:100, Cedarlane, PM045, Dallas, TX, USA). Cells were washed three times with PBS and incubated with goat anti-rabbit AlexaFluor 594 (1:500, #A-11012, FisherSci, Ottawa, ON, Canada) or goat-anti mouse AlexaFlour 594 (1:500, #A-11011, FischerSci, Ottawa, ON, Canada). Cells were then washed three times with PBS for 5 min and then incubated with DAPI (1:2000) for an additional five minutes. The 96-well μ-PLATE square ibiTreat plates were then imaged using the Opera high content imaging system and analyzed by Columbus™ software (Version 2.5) to measure Srgn and p62 puncta size and area.

### 2.9. Lysis of Cells

Cell lysates and skeletal muscle homogenates were prepared with radioimmunoprecipitation assay (RIPA) buffer (150 mM NaCl, 50 mM Tris-HCl (pH 8), 1% Triton X-100, 0.5% NaDOC, 0.1% SDS) supplemented with protease (Roche, 04693116001, Mississauga, ON, Canada) and phosphatase (Roche, PHOSS-RO) inhibitors. Cell lysates were centrifuged at 15,000× *g* for 10 min at 4 °C, and the supernatants were collected. Skeletal muscle samples were combined with lysis buffer and a metal bead in 2 mL SafeLock™ Eppendorf tubes (Eppendorf, 022363352, Mississauga, ON, Canada), then homogenized using a TissueLyser II (Qiagen, Toronto, ON, Canada). The protocol comprises 3 rounds of homogenization at 30 Hz for 90 s, followed by incubation on ice for 60 s. Afterwards, samples were kept at 4 °C for 4 h on a rotating platform, then centrifuged at 15,000× *g* for 15 min at 4 °C. Total protein concentration was determined using a DC Protein Assay (BioRad, 5000111, Toronto, ON, Canada) with BSA dilutions (Pierce, 23209, Toronto, ON, Canada) as standards.

### 2.10. Conditioned Medium Collection for Srgn Secretion

To examine Srgn protein levels within conditioned medium, C2C12 cells were seeded in 10 cm plates. *Gfpt1* silencing was achieved by doxycycline treatment at 2 μg/mL for 72 h. (Sigma, D9891, Toronto, ON, Canada). Cell media was replaced with serum-starved growth medium for 1 h. Afterwards, the growth medium was reapplied and incubated for 1, 3, 5, 8, 12, 18, and 24 h. At each timepoint, cell media was collected and combined in a 1:5 ratio with ice-cold 100% methanol and placed in the −80 °C overnight. The next morning, the solution was centrifuged at 8000× *g* for 30 min. The supernatant was discarded, and pellets were dried for 1 h in a fume hood. The cell pellet was homogenized with RIPA buffer, followed by a brief sonification.

### 2.11. Western Blotting

To examine protein levels within *Gfpt1*-deficient cells and skeletal muscle, lysates were resolved by SDS-PAGE and transferred to PVDF membranes using a BioRad™ Trans-Blot Turbo system (1704150EDU, Mississauga, ON, Canada). All samples were heated at 100 °C for 5 min in 4× Laemmli sample buffer, prior to resolution with Western blot. When examining Srgn protein levels, SDS-PAGE gels were transferred to PVDF membranes with wet transfer (40 V, overnight, 4 °C). All membranes were blocked for 1 h and incubated with primary antibody overnight at 4 °C. Multiple secondary antibodies were used for this analysis, including IR Dye^®^800CW Donkey anti-Mouse IgG (Li-Cor, 926-32212, 1:5000, Lincoln, NB, USA) and IR Dye^®^680RD Goat anti-Rabbit IgG (Li-Cor, 926-68071, 1:5000) for 1 h at room temperature.

Blots were imaged using a Licor Odyssey DLX (Lincoln, NE, USA) and densitometry performed using Image Studio™ (Licor, version 6.0) or with ChemiDoc (BioRad, Mississauga, ON, Canada) and analyzed with ImageJ (Version 1.54). Gapdh or Ponceau S stain was used to normalize protein expression.

### 2.12. Real-Time qPCR (RT-qPCR)

RNA collection for in vitro experiments was performed using the RNeasy™ Micro mini kit (Qiagen, #74134, Toronto, ON, Canada). Skeletal muscle RNA extraction was performed with the RNeasy™ Fibrous Micro mini kit (Qiagen, #74704) and quantified by NanoDrop (ND-1000, Spectrophotometer, NanoDrop^®^, Wilmington, DE, USA). RNA was converted to complementary DNA (cDNA) with the All-In-One 5x RT MasterMix (ABM, #G592, Richmond, BC, Canada) following kit guidelines. RT-qPCR was performed using the PowerUp™ SYBR™ Green Master Mix (FisherSci, #A25741, Ottawa, ON, Canada) with the QuantStudio™ Studio 6 Flex Real-Time PCR System (Thermofisher, Ottawa, ON, Canada) (see Supplementary Materials Table S1 for primer sequences). To analyze the expression of our target genes, the geometric mean of three reference genes (*Gapdh*, *Rpl27*, and *Ppia*) was used.

### 2.13. Soluble/Insoluble Fractionation

*Gfpt1*-deficient and scramble myoblasts were seeded in 10 cm plates. Following activation for gene silencing with 2 μg/mL doxycycline for 72 h, the cells were pelleted and resuspended in cell lysis buffer (20 mM HEPES, pH 7.9, 0.2 M KCl, 1 mM MgCl_2_, 1 mM EGTA, 1% Triton X-100, 10% glycerol, and protease/phosphatase inhibitor) and incubated on ice for 30 min. The soluble fraction was taken from the supernatant, which was collected after centrifugation at 13,000× *g* for 20 min at 4 °C. The pellet (insoluble membrane) was solubilized in an SDS-detergent buffer (20 mM HEPES, pH 7.9, 0.2 M KCl, 1 mM MgCl_2_, 1 mM EGTA, 1% Triton X-100, 1% SDS, 10% glycerol and protease/phosphatase inhibitor). Cell pellets were solubilized using a mortar and pestle. All samples were heated at 100 °C for 5 min in 4× Laemmli Sample Buffer, prior to resolution with Western blot.

### 2.14. Antibodies

The following antibodies were used for immunoblot or immunofluorescent experiments: Gfpt1 (ProteinTech, #14132-1-AP, 1:500, Rosemont, IL, USA), Gapdh (14C10) (Cell Signalling, 2118, 1:2000, Danvers, MA, USA), O-GlcNAc (RL2) (Novus Biologicals, NB300-524, 1:1000, Toronto, ON, Canada), Ogt (ProteinTech, 66823-1-Ig, 1:1000, Rosemont, IL, USA), Srgn (#C-11, Santa Cruz Technology, Dallas, TX, USA), Srgn (#H9, Santa Cruz), p62 (Cedarlane, #PM045), LC3 (Sigma, #L7543).

### 2.15. Statistics

Data was analyzed with GraphPad Prism v 10.1.2. Data was checked for normality using the D’Agostino-Pearson test and outliers were identified using the robust regression and outlier remover (ROUT) method. Statistical significance was determined by using unpaired two-tailed Student *t*-tests or analysis of variance (ANOVA) tests. If multiple comparisons were used, Tukey’s post hoc test was performed. Results are presented as mean ± SD, where *p* < 0.05 was considered significant.

## 3. Results

### 3.1. Whole-Cell Proteomics in Gfpt1-Deficient Myoblasts Reveals Dysregulation of Autophagy and Intracellular Trafficking Pathways

**Figure 1:**
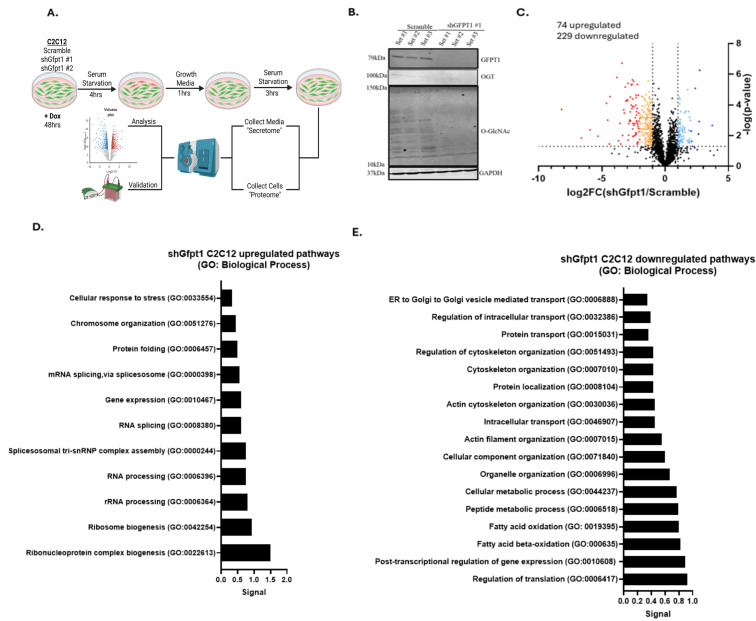
Whole-cell proteomics in *Gfpt1*-deficient myoblasts reveals impaired autophagy and protein trafficking: (**A**). A schematic of the proteomic (cell pellets) and secretomic (conditioned medium) isolated from *Gfpt1*-deficient myoblasts. The cell pellets (containing all possible membrane, cytosolic, and nuclear proteins) were harvested and snap-frozen in liquid nitrogen for downstream proteomic analysis of both conditioned media and whole-cell lysates. (**B**). Immunoblot analysis performed prior to proteomic assessment confirms reduced Gfpt1 protein levels and decreased global O-GlcNAcylation in doxycycline-treated myoblast lysates. Samples were isolated from three independent biological experiments. (**C**). Volcano plot of whole-cell proteomics comparing *Gfpt1*-deficient and scramble control C2C12 myoblasts identifies 74 upregulated and 220 downregulated proteins. Differential expression was defined as a fold change ≥ 2.0 or ≤0.5 with statistical significance (*p* < 0.05). (**D**). STRING pathway analysis was performed on upregulated proteins and (**E**). downregulated proteins. For both conditions, the top 15 pathways implicated with *Gfpt1*-deficient myoblasts. The size of the circle for each pathway indicates the number of proteins implicated in each respective pathway. Original images can be found in Supplementary File S1.

To define the global proteomic consequences of reduced hexosamine biosynthetic pathway flux, we performed whole-cell mass spectroscopy on both the conditioned medium (secretome) and cell pellets (proteome) of doxycycline-inducible *Gfpt1*-deficient C2C12 myoblasts (Figure 1A). Prior to proteomic profiling, we confirmed efficient knockdown of Gfpt1, oligotransferase (Ogt), and a decrease in global O-GlcNAcylation by Western blotting, which is consistent with diminished HBP flux (Figure 1B).

Comparative proteomic analysis between *Gfpt1*-deficient and scramble myoblasts identified 74 significantly upregulated and 229 significantly downregulated proteins (Figure 1C). Our comparative analysis revealed that Stead3 (6.65-fold), Slc25a10 (6.45-fold), Rab34 (6.23-fold), Rhob (5.20-fold), and Supt16h (4.50-fold) had the highest magnitude of change (Table 1). The magnitude and directionality of these broad changes suggest widespread alterations in proteostasis and cellular responses. STRING pathway enrichment analysis of upregulated proteins demonstrated prominent activation of pathways relevant to protein homeostasis, protein folding, and stress-responsive chaperone systems (Figure 1D). These findings are consistent with an adaptive response to impaired glycosylation and an increased burden of misfolded or immature proteins.

In contrast, our comparative analysis revealed that Ppp1r14b (0.02-fold), Lima1 (0.04-fold), Gmfb (0.05-fold), Cttn (0.07-fold), and Denr (0.08-fold) represent the most downregulated proteins (Table 1). Overall, downregulated proteins showed a strong enrichment for pathways associated with intracellular transport, including vesicle-mediated trafficking, and protein processing in the secretory pathway (Figure 1E). Along this line, several components of COPII-mediated ER-to-Golgi transport machinery, Golgi organization, and vesicle cargo sorting systems were reduced, suggesting compromised protein trafficking capacity (Table 1). Together, these proteomic changes indicate that *Gfpt1* deficiency disrupts the balance between protein synthesis, folding, and notably trafficking, resulting in the coordinated activation of stress-mitigating pathways and suppression of secretory transport machinery.

### 3.2. Gfpt1 Deficiency Activates UPR Through Increased Xbp1 Splicing and Atf4

**Figure 2:**
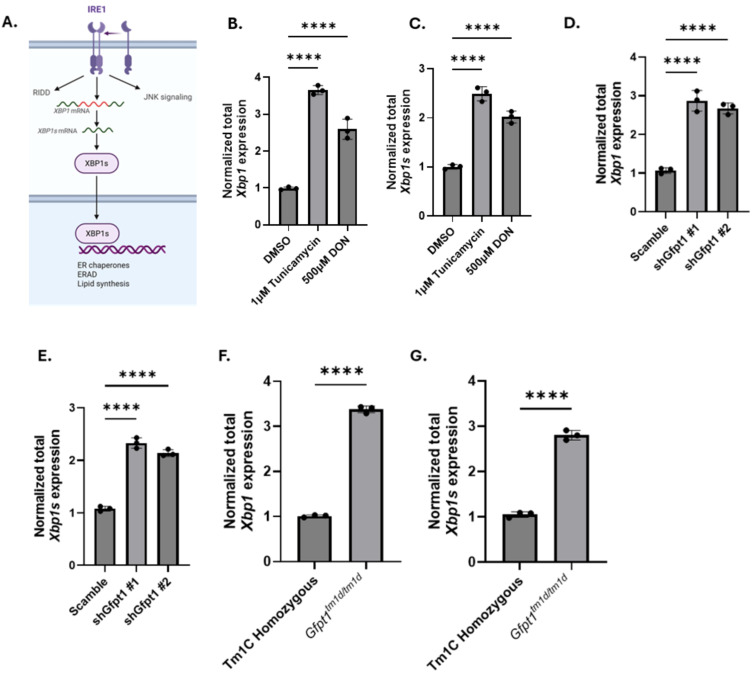
*Gfpt1*-deficient models show elevated levels of *Xbp1s* expression, indicating activation of the unfolded protein response. (**A**). Schematic illustration of the IRE1-XBP1 arm of the unfolded protein response (UPR). *Gfpt1* deficiency reduces protein glycosylation, resulting in a hypoglycosylated cellular environment. To prevent the accumulation of misfolded proteins, cells activate UPR pathways that regulate protein folding and degradation. Under ER stress, X-box binding protein 1 (XBP1) undergoes IRE1-dependent alternative splicing to generate *XBP1s*, a transcription factor that induces expression of ERAD components and ER chaperones. (**B**). C2C12 myoblasts were treated with tunicamycin or 6-diazo-5-oxo-L-norleucine (DON) to induce hypoglycosylation. RT-qPCR analysis shows increased expression of total *Xbp1* and (**C**). spliced *Xbp1* (*Xbp1s*). (**D**). Doxycycline-treated *Gfpt1*-deficient C2C12 myoblasts (shGfpt1 #1 and shGfpt1 #2) exhibit increased expression of total *Xbp1* and (**E**). *Xbp1s* relative to scramble controls. (**F**). RT-qPCR analysis of quadriceps muscle from *Gfpt1^tm1d/tm1d^* mice and Tm1C homozygous control mice shows increased total *Xbp1* expression and (**G**). elevated *Xbp1s* expression in *Gfpt1*-deficient samples. Gene expression values were normalized to the geometric mean of *Rpl27*, *Gapdh*, and *Actb*. Three biological replicates were analyzed for all experiments. Statistical significance was determined by one-way ANOVA (**B**–**E**) or Student’s *t*-test (**F**,**G**). **** *p* < 0.0001.

Our proteomic results revealed many proteins associated with trafficking and ER homeostasis. As a result, in elevated hypoglycosylation stress, we hypothesized that the unfolded protein response (UPR) pathway would also be impaired. The UPR consists of three coordinated signaling branches—IRE1, PERK and ATF6—that collectively restore ER proteostasis by enhancing chaperone expression, attenuating protein synthesis, and promoting ER-associated degradation (ERAD) (Figure 2A). Under ER stress, the IRE1 arm generates the active transcription factor Xbp1s via unconventional mRNA splicing, thereby increasing the transcription of ERAD components and ER chaperones. XBP1 exists in two forms: an unspliced, relatively unstable transcript (*Xbp1u*) and a spliced form (*Xbp1s*) produced during ER stress (Figure 2A). Consistent with activation of this pathway, we observed increased levels of *Xbp1s* accompanied by a relative reduction in *Xbp1u*, indicating enhanced IRE1 signaling and unfolded protein response induction.

To determine whether hypoglycosylation is sufficient to activate Xbp1, we treated C2C12 cells with increasing doses of tunicamycin, an inhibitor of N-linked glycosylation. We found a significant dose-dependent increase in total *Xbp1*, *Xbp1s*, and a decrease in *Xbp1u* expression (Supplementary Figure S1A–C). We next aimed to examine whether pharmacological inhibition of Gfpt1 with 6-diazo-l-norleuncine (DON) could activate *Xbp1* expression. Indeed, DON inhibition of Gfpt1 significantly increased total *Xbp1* transcript levels, robustly elevated *Xbp1s* and decreased *Xbp1u* expression levels (Figure 2A,B, Supplementary Figure S1D). As such, both inhibition of N-linked glycosylation and Gfpt1 directly engage the IRE1 pathway in response to hypoglycosylation stress.

We next examined whether *Gfpt1* knockdown directly activates the UPR. Doxycycline-inducible *Gfpt1*-deficient myoblasts (shGfpt1 #1 and shGfpt1 #2) displayed significantly elevated expression of both total *Xbp1* and *Xbp1s* relative to scramble controls (Figure 2D,E). There was a significant decrease in *Xbp1u* expression levels (Supplementary Figure S1E). As such, this demonstrates that reduced HBP flux through *Gfpt1* deficiency activates the IRE1–*Xbp1* axis.

To evaluate whether this response also occurs in vivo, we assessed ER stress markers in whole skeletal muscle isolates from 20-week *Gfpt1^tm1d/tm1d^* mice. *Gfpt1^tm1d/tm1d^* mice harbor a conditional skeletal muscle-specific knockout of *Gfpt1*, which contains a pathological resemblance to *GFPT1*-CMS patients, and has been used to test the efficacy of new therapeutic strategies. Quadriceps muscle from *Gfpt1^tm1d/tm1d^* mice exhibited significantly increased total *Xbp1* and *Xbp1s* expression compared to Tm1C homozygous control mice (Figure 2F,G). We also highlighted a significant decrease in *Xbp1u*, confirming activation of the UPR in *Gfpt1^tm1d/tm1d^* mice (Supplementary Figure S1F).

Together, these findings demonstrate that *Gfpt1* deficiency consistently activates the IRE1-*Xbp1* branch of the UPR across *Gfpt1*-deficient myoblasts and skeletal muscle. This indicates that impaired glycosylation creates sufficient proteotoxic stress to engage ER-adaptive pathways.

### 3.3. Gfpt1-Deficiency Impairs Autophagy and Leads to p62 Accumulation in Myoblasts and Skeletal Muscle

Since our proteomics and secretion analyses suggested broad disruption in proteostasis, we next examined whether *Gfpt1*-deficiency alters autophagy, a major pathway responsible for clearing misfolded and aggregated proteins. Notably, whole-cell proteomics identified multiple autophagy-related proteins as dysregulated in *Gfpt1*-deficient myoblasts, including components involved in cargo recognition, autophagosome formation, and lysosomal turnover (summarized in Table 1).

**Figure 3:**
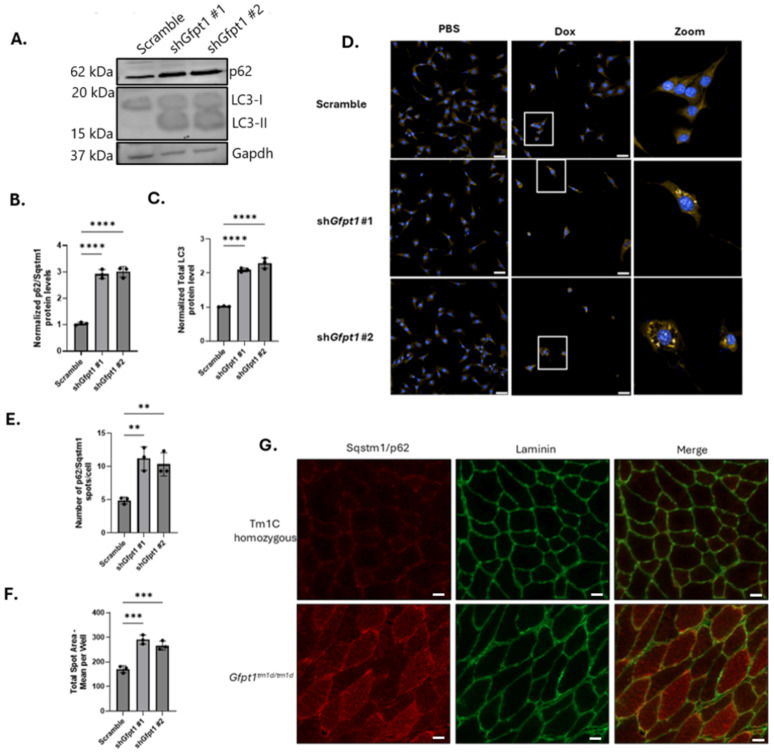
Whole-cell proteomics in *Gfpt1*-deficient myoblasts reveals impaired autophagy (**A**). Western blot analysis shows increased p62 and LC3-II protein levels in *Gfpt1*-deficient myoblasts compared with scramble controls. (**B**). Quantification of immunoblot data reveals a significant increase in p62 and (**C**). LC3-II protein levels in *Gfpt1*-deficient myoblasts. (**D**). Immunofluorescence analysis demonstrates increased p62 staining intensity in *Gfpt1*-deficient myoblasts. (**E**). Quantification of autophagic markers shows a significantly higher percentage of cells containing p62-positive puncta and (**F**). an increase in p62 puncta area in *Gfpt1*-deficient myoblasts, indicative of impaired autophagic flux. (**G**). Immunofluorescence staining of p62 in gastrocnemius muscle from *Gfpt1^tm1d/tm1d^* mice reveals increased signal intensity and punctate accumulation within muscle fibers. Muscle fibers were marked with α1 laminin (Lama1). Scale bar: 20 µm. All graphs represent mean ± SD. Statistical significance was assessed using one-way ANOVA. All experiments were performed with three biological replicates per group. Comparisons were made between scramble control and *Gfpt1*-deficient samples. ** *p* < 0.01, *** *p* < 0.001 and **** *p* < 0.0001. Original images can be found in Supplementary File S1.

To validate these findings, we performed biochemical analyses in doxycycline-induced *Gfpt1*-deficient C2C12 myoblasts. Immunoblotting showed a robust accumulation of p62 and increased LC3-II levels compared with scramble controls (Figure 3A), and densitometric quantification confirmed significant elevations in both proteins (Figure 3B and Figure 3C, respectively). To further dissect p62 behavior, we performed soluble/insoluble fractionation. In control cells, p62 was primarily detected in the soluble fraction; however, *Gfpt1*-deficient myoblasts displayed marked accumulation of p62 in both soluble and insoluble fractions (Supplementary Figure S2A). Insoluble p62 is a biochemical hallmark of defective autophagic flux and protein aggregation, further supporting a block in cargo clearance [30].

To determine whether *Gfpt1* deficiency affects autophagic degradation rather than initiation, we inhibited lysosomal acidification with chloroquine. In C2C12 cells, chloroquine treatment increased p62 staining intensity (Supplementary Figure S2B,C). Notably, *Gfpt1*-deficient myoblasts displayed elevated p62 levels following chloroquine treatment (Supplementary Figure S2D,E). We next assessed whether *Gfpt1* deficiency exacerbates inhibition of autophagic flux. Consistent with the biochemical data, immunofluorescence analysis revealed increased p62 signal intensity in *Gfpt1*-deficient myoblasts (Figure 3D). Quantitative image analysis revealed a significantly increased number of p62-positive puncta (Figure 3E) and area (Figure 3F), respectively. These results suggest that there may be an accumulation of autophagic cargo.

Next, we explored whether p62 puncta accumulated within *Gfpt1^tm1d/tm1d^* mice’s skeletal muscle. Previous whole-cell proteomics from *Gfpt1^tm1d/tm1d^* revealed a ~2.5-fold increase in p62 protein levels in intercostal muscles [22]. To confirm this proteomic result, we performed p62 staining within the gastrocnemius muscle isolated from *Gfpt1^tm1d/tm1d^* and Tm1C homozygous muscle. We demonstrate that indeed p62-positive puncta accumulated within the gastrocnemius muscle, further demonstrating that dysfunctional autophagy extends to the skeletal muscle (Figure 3G).

Together, these findings demonstrate that *Gfpt1*-deficiency leads to persistent accumulation of p62, elevated LC3-II, and increased puncta formation. This autophagy defect is consistent with the pronounced proteostasis and trafficking abnormalities observed in *Gfpt1*-deficient myoblasts and *Gfpt1^tm1d/tm1d^* skeletal muscle.

### 3.4. Gfpt1 Deficiency Impairs Protein Trafficking and Reduces Secretion of Skeletal Muscle Proteoglycan Serglycin

Our whole-cell proteomic analysis indicated suppression of intracellular trafficking pathways in *Gfpt1*-deficient myoblasts. This led us to examine whether defects in protein transport affect the secretion of muscle-derived cargo proteins. Secretomic profiling detected 140 proteins in conditioned medium, with 13 proteins significantly altered in *Gfpt1*-deficient C2C12 myoblasts. (Table 2). Among stress-associated proteins, we observed a pronounced reduction in the extracellular abundance of Binding Immunoglobulin Protein (BiP) in *Gfpt1*-deficient C2C12 cells (Table 2). Reduced BiP secretion likely reflects increased ER retention due to elevated chaperone engagement with misfolded proteins, together with impaired ER-to-Golgi trafficking obtained from the proteomics of *Gfpt1*-deficient myoblasts.

**Figure 4:**
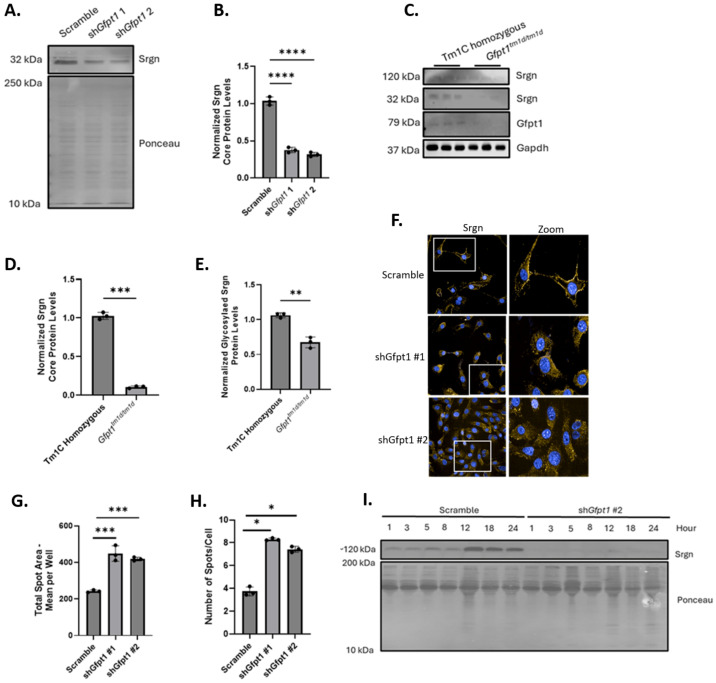
Proteomics reveals downregulation in protein trafficking pathways and altered secretion of skeletal muscle proteins. (**A**). Western blot analysis of serglycin (Srgn) protein levels in *Gfpt1*-deficient C2C12 myoblasts. Protein levels were normalized to total protein loading using Ponceau S staining. (**B**). Quantification shows a significant reduction in Srgn protein levels in *Gfpt1*-deficient myoblasts using two independent shRNA constructs. (**C**). Western blot analysis of quadriceps muscle lysates from 20-week-old *Gfpt1^tm1d/tm1d^* mice and Tm1C homozygous controls, showing high-molecular-weight (~120 kDa) glycosylated Srgn species, core (~32 kDa) Srgn, and Gfpt1. (**D**). Quantification of core Srgn and (**E**). high-molecular-weight glycosylated Srgn species, normalized to Gapdh protein levels. (**F**). Immunofluorescence staining of scramble control and *Gfpt1*-deficient C2C12 myoblasts labeled for Srgn. Images were acquired at 40× magnification using the OPERA™ high-content imaging system. Scale bar = 10 µm. (**G**). Quantitative image analysis using Columbus™ software shows increased total Srgn-positive spot area (µm^2^) and (**H**). an increased average number of Srgn-positive puncta per cell in *Gfpt1*-deficient myoblasts. (**I**). Immunoblot analysis of conditioned medium collected over a 24 h time course from scramble control and shGfpt1 #2 C2C12 myoblasts. All graphs represent mean ± SD. Statistical significance was assessed using one-way ANOVA. All experiments were performed with three biological replicates per group. * *p* < 0.05, ** *p* < 0.01, *** *p* < 0.001, **** *p* < 0.0001. Original images can be found in Supplementary File S1.

Serglycin (Srgn) is a secreted proteoglycan with roles in skeletal muscle biology. Given that our proteomic and secretomic analyses suggested impaired protein trafficking, we next investigated whether Srgn secretion is affected by *Gfpt1* deficiency. In this context, impaired glycosylation may disrupt proper Srgn processing and export, thereby impacting muscle homeostasis. Immunoblot analysis of doxycycline-induced *Gfpt1*-deficient C2C12 cells demonstrated a marked reduction in Srgn protein levels in both shGfpt1 #1 and shGfpt1 #2 lines, normalized to Ponceau S staining (Figure 4A,B).

To determine whether Srgn dysregulation occurs in vivo, we analyzed quadriceps from muscle from 20-week-old *Gfpt1^tm1d/tm1d^* mice. Consistent with the in vitro results, *Gfpt1*-deficient quadriceps exhibited reduced levels of both the core Srgn protein (~32 kDa) and the high molecular weight glycosylated Srgn species (~120 kDa) (Figure 4C,E). Together, these results indicate that *Gfpt1*-deficiency alters the abundance of Srgn.

Immunofluorescence microscopy revealed an abnormal intracellular distribution of Srgn in *Gfpt1*-deficient C2C12 myoblasts. Compared with scramble controls, where Srgn was predominantly localized in subsarcolemmal regions, *Gfpt1*-deficient cells exhibited increased cytoplasmic Srgn-positive puncta and enhanced intracellular staining intensity (Figure 4F). Quantitative image analysis using Columbus™ software showed significant increases in both the total Srgn-positive spot area (Figure 4G) and the number of Srgn-positive puncta per cell (Figure 4H), indicating intracellular accumulation. Taken together, these data indicate that overall Srgn levels are reduced in *Gfpt1*-deficient myoblasts. However, the remaining Srgn is predominantly retained intracellularly, as opposed to being membrane-bound and secreted.

Finally, analysis of conditioned medium collected over a 24 h time course revealed a reduction in secreted Srgn from *Gfpt1*-deficient myoblasts relative to scramble controls (Figure 4I and Supplementary Figure S3A). Consistently, reduced total Srgn levels were observed at 24 h (Supplementary Figure S3B,C). Together, these findings indicate that *Gfpt1*-deficiency disrupts intracellular protein trafficking, leading to retention and decreased secretion of Srgn in murine myoblasts.

## 4. Discussion

GFPT1 is the rate-limiting enzyme of the HBP, generating UDP-GlcNAc, the obligate donor substrate for multiple forms of protein glycosylation. Since the HBP sits at the interface of nutrient sensing, protein quality control and membrane trafficking, disruptions in GFPT1 activity, as evident in *GFPT1*-CMS, are expected to impose broad metabolic and proteostatic consequences on muscle cells [4,5]. In this study, we demonstrate that loss of Gfpt1 leads to coordinated disturbances of glycosylation-dependent proteostasic mechanisms, spanning ER stress, vesicle trafficking, secretion and autophagy. This provides mechanistic insights into the skeletal muscle pathology observed in *GFPT1*-CMS patients.

*GFPT1*-CMS is characterized by fatigable muscle weakness caused by biallelic mutations that reduce GFPT1 protein abundance and enzymatic levels [10,14,18,19,20,21]. We previously showed that impaired Gfpt1 function disrupts protein glycosylation, leading to hypoglycosylation of AChRδ, a defect that can be rescued by galactose supplementation [6,23]. This hypoglycosylated environment may also contribute to the formation of tubular aggregates, a shared feature of other glycosylation-related CMS subtypes (GMPPB, ALG2, ALG14, and DPAGT1), highlighting the need to define upstream drivers of pathology [11,12,13,32,33,34,35].

Consistent with this, we observed robust activation of IRE1-XBP1 arms of the UPR in both pharmacologic and genetic models of *Gfpt1*-deficiency [26,36]. Given the sensitivity of IRE1 signaling to defects in glycan maturation, these findings indicate that ER stress arises as a primary consequence of impaired HBP flux rather than secondary degeneration [37,38]. This finding is consistent with other myopathies driven by glycosylation defects, such as GNE myopathy and congenital disorders of glycosylation (CDGs), where early pathological features depict UPR activation and ER stress [39,40,41,42,43,44,45,46].

A significant finding of this study is the global downregulation of vesicle trafficking pathways. Proteomic analysis revealed an abundance of COP-I/COP-II components, ER–Golgi transport factors, and proteins involved in vesicle formation and cargo sorting. As these processes depend on proper glycosylation for protein stability, folding, and export, these data suggest a multi-level disruption of secretory pathway function [1,3,47]. This is functionally supported by impaired secretion of Srgn, a highly glycosylated proteoglycan requiring intact ER–Golgi processing for maturation and export. Its intracellular punctate accumulation alongside reduced secretion indicates a failure of secretory machinery and highlights a broader defect in extracellular matrix (ECM) protein handling [48].

These changes could have negative consequences for skeletal muscle health [3]. Secretory defects may affect skeletal muscle health because myofibers rely heavily on efficient export of ECM proteins, proteoglycans, and signaling factors to maintain structural stability and intercellular communication [49,50]. Importantly, Srgn knockout mice develop normally and do not display an overt skeletal muscle phenotype, suggesting that Srgn itself is not essential for muscle development under basal conditions [51]. Therefore, the reduction and intracellular accumulation of Srgn observed in our models likely reflect impaired glycosylation-dependent trafficking rather than a primary pathogenic driver. In this context, Srgn may serve as a sensitive marker for secretory pathway dysfunction, highlighting broader defects in protein export that could ultimately compromise muscle homeostasis. A potential mechanism of impact could be through immunological responses, which have been shown in Srgn-deficient mouse models [52]. Importantly, similar defects in proteoglycan secretion have been identified in other muscle disorders, and CDGs, all of which exhibit impaired extracellular matrix assembly, disrupted sarcolemmal stability, and progressive myofiber degeneration [53,54,55]. As such, examination of additional proteoglycan synthesis, packaging, and trafficking should be extensively studied within *Gfpt1*-deficient models.

It is important to note that while Srgn was not detected within the secretomic profiling of conditioned medium from *Gfpt1*-deficient myoblasts, this likely reflects inherent limitations in mass spectroscopy-based detection of heavily glycosylated proteoglycans rather than true absence [56,57]. Consistent with this, immunoblot analysis confirmed reduced secretion and intracellular accumulation of Srgn, supporting a defect in glycosylation-dependent trafficking. Together, these findings suggest that Srgn may represent a sensitive marker of impaired secretory pathway function in the context of *Gfpt1* deficiency.

However, secretomic profiling of *Gfpt1*-deficient myoblasts did reveal a secreted-BiP deficiency. BiP is a central regulator of ER proteostasis and secretory capacity as it supports folding of nascent luminal proteins, enforces ER quality control, and maintains export-competent cargo; thus, reduced BiP is expected to constrain secretion by promoting ER retention, ERAD, and stress-induced translational attenuation [58,59]. This defect is exacerbated by impaired glycosylation in *Gfpt1*-deficient cells, as glycan modification is critical for protein stability, chaperone engagement, and trafficking [1,6,60]. Consistently, we observed reduced secretion of Srgn, a highly glycosylated proteoglycan that serves as a sensitive readout of ER quality control.

Although BiP is primarily an ER-resident protein, it can also be secreted when it participates in stress signaling. BiP secretion has been reported to decrease under ER stress, including tunicamycin treatment, suggesting a role in adaptive intercellular communication [61,62,63]. The reduction observed here therefore indicates not only impaired folding capacity but also defective stress-responsive secretion. Given BiP’s central role in regulating the UPR, reduced intracellular and secreted BiP may limit the ability to buffer ER stress in *Gfpt1*-deficient myoblasts [58,61]. Moreover, extracellular BiP has been linked to cytoskeletal regulation and neurite outgrowth, raising the possibility that its loss affects stress resilience and cellular remodeling [64]. Together, impaired glycosylation, reduced BiP, and defective secretion of Srgn support a model in which ER proteostasis failure manifests as both intracellular folding insufficiency and loss of adaptive secretory signaling.

In parallel, *Gfpt1*-depleted myoblasts show increased release of cytosolic proteins consistent with stress-induced unconventional secretion during proteotoxic states [65,66]. This shift from classical ER–Golgi trafficking to noncanonical export may reflect membrane instability, impaired vesicle maturation or increased extracellular vesicle release [67,68]. Similar secretory remodeling has been observed in myopathies associated with ER stress or impaired autophagy, reinforcing the idea that proteostasis disruption fundamentally reshapes the muscle secretome [69,70].

Given that the NMJ is highly dependent on efficient secretion, defects in intracellular trafficking provide a direct mechanistic link to CMS pathology. NMJ maintenance requires continuous export of synaptic organizers, including agrin, laminin α2/β2, perlecan, and collagens, as well as trafficking of AChR complexes and associated signaling factors [71,72,73]. These processes rely on an intact ER–Golgi transport and glycosylation-dependent cargo maturation. Consistent with this, reduced AChR surface has been detected in vitro, along with decreased α-BTX staining at the NMJs in *Gfpt1^tm1d/tm1d^* muscle. Previously, AChR subunits were found to have reduced surface expression in transfected HEK293T cells [14]. In addition, previous data show reduced α-BTX staining at the NMJ of *Gfpt1^tm1d/tm1d^*, suggesting impairment to the surface expression and/or stability of the AChR complex [6,23]. Thus, trafficking defects observed in our models, including Srgn retention, likely impair the delivery of essential synaptic proteins and chaperones such as BiP. In this context, impaired secretion may represent a central pathogenic mechanism, weakening synaptic integrity and increasing NMJ susceptibility to stress [7,74,75,76,77].

We also demonstrate defects in autophagic clearance, likely downstream of ER stress and trafficking disruption. Autophagy requires coordinated membrane trafficking, ER–Golgi dynamics, and lipidation, processes dependent on intact glycosylation pathways [27,29]. In *Gfpt1*-deficient myoblasts, elevated p62 and LC3-II levels indicate impaired autophagic flux rather than increased initiation, a finding recapitulated in *Gfpt1^tm1d/tm1d^* muscle [78,79,80].

This aligns with prior reports showing enhanced protein aggregation and reduced autophagy following *Gfpt1* silencing [81]. Thus, protein aggregation and impaired autophagy may contribute to muscle fiber pathology in *GFPT1*-CMS.

In addition, future studies should aim to further define the mechanistic links between impaired HBP flux and defects in protein trafficking, secretion and autophagy. Previous work demonstrated that metabolic supplementation with galactose has been shown to improve skeletal muscle health, NMJ morphology, and neurotransmission in *Gfpt1^tm1d/tm1d^* models, making it of particular interest to determine whether the proteostasis and secretory defects observed in this study could be rescued through similar approaches [23]. In addition, building on the identification of hypoglycosylated AChRδ, further investigation of NMJ integrity in the context of disrupted trafficking may help clarify the contribution of these pathways to disease pathology. Finally, targeted analysis of extracellular matrix components and secreted proteoglycans may help establish whether molecules such as Srgn could serve as a biomarker or therapeutic targets in *GFPT1*-CMS.

## 5. Conclusions

In summary, this study demonstrates that *Gfpt1* deficiency disrupts glycosylation-dependent proteostasis in skeletal muscle, leading to broad alterations in ER homeostasis, intracellular trafficking, secretion and autophagic flux. Reduced HBP flux triggers activation of the IRE1-XBP1 branch of the UPR, while impairing vesicular transport and secretory capacity. These defects result in intracellular retention and reduced secretion of key glycoproteins, including proteoglycan Srgn, highlighting disruption of protein secretion. Importantly, these interconnected defects provide a unifying mechanistic framework linking impaired glycosylation to NMJ dysfunction, reduced stress resilience, and skeletal muscle pathology in *GFPT1*-CMS. Overall, this work identifies glycosylation-dependent proteostasis as a central determinant of muscle integrity and highlights intracellular trafficking and secretory dysfunction as key contributors to disease pathogenesis.

## Figures and Tables

**Table 1:** Key proteins up- or downregulated in *Gfpt1*-deficient C2C12 myoblasts compared with scramble. Proteins with a *p*-value of <0.05 and an abundance ratio of either ≥2-fold (upregulated) or ≤0.5-fold (downregulated) are included below.

Protein	Description	Autophagy-Relevant Pathway Role	KD/Ctrl
Map1lc3b	LC3B, autophagosome membrane protein	Core macroautophagy machinery; autophagosome formation, mitophagy, pexophagy	2.25
Rheb	mTORC1 activator GTPase	Master negative regulator of autophagy via mTORC1	4.17
Rab34	Rab GTPase	Lysosome positioning; autophagosome–lysosome fusion	6.23
Rhob	Rho GTPase	Endosomal/lysosomal trafficking; autophagosome maturation	5.20
Emc6	ER membrane complex subunit	ER-derived phagophore membrane source	2.36
Calr	ER Ca^2+^ chaperone	ER stress, ER-phagy, autophagy induction	2.19
Erp29	ER folding protein	UPR/ER stress-induced autophagy	2.18
Erlin2	ERAD/ER stress protein	ER stress signaling to autophagy	2.17
Psmb3	Proteasome subunit	Proteasome–autophagy compensatory axis	2.35
Hspbp1	Hsp70 co-chaperone	Chaperone-assisted autophagy/proteostasis	2.11
Ppib	ER peptidyl-prolyl isomerase	ER proteostasis → autophagy induction	2.40
Clcn5	Endolysosomal Cl^−^ channel	Lysosome acidification; autophagic degradation	2.63
Arsb	Lysosomal enzyme	Lysosomal throughput during autophagy	2.31
Ndufs1	ETC Complex I subunit	Mitophagy cargo indicator	3.80
Ndufa6	ETC Complex I subunit	Mitophagy cargo indicator	2.27
Mtco2	ETC Complex IV subunit	Mitophagy cargo indicator	2.27
Cox6c	ETC Complex IV subunit	Mitophagy cargo indicator	2.15
Bcat1	BCAA metabolism enzyme	Nutrient sensing → mTOR regulation	2.52
Slc25a10	Mitochondrial carrier	Redox/TCA control → AMPK/mTOR/autophagy	6.45
Ampd2	AMP metabolism enzyme	AMP/ATP ratio → AMPK → autophagy	2.61
Copg1	Coatomer subunit gamma-1	COPI vesicle trafficking (Golgi ↔ ER retrograde transport)	0.50
Sec23a	COPII vesicle component	ER → Golgi export (cargo sorting)	0.49
Sec22b	SNARE protein	ER–Golgi vesicle fusion, ER-phagosome pathway	0.31
Uso1	Vesicle tethering factor (p115)	Golgi vesicle docking and fusion	0.42
Rab5b	Small GTPase	Early endosome formation, endocytosis	0.44
Snx9	Sorting nexin-9	Clathrin-mediated endocytosis	0.46
Gapvd1	Rab5 GEF	Endosome maturation and trafficking	0.43
Clta	Clathrin light chain A	Vesicle coat formation (endocytosis)	0.25
Prkaa1 (AMPKα1)	Energy sensor kinase	Autophagy induction, mTOR regulation	0.42
Hspb8	Small heat shock protein	BAG3-mediated selective autophagy (aggrephagy)	0.42
Bag3	Co-chaperone	Protein quality control, autophagy targeting	0.12
Stub1 (CHIP)	E3 ubiquitin ligase	Proteasome targeting + autophagy crosstalk	0.43
Psmb7	Proteasome subunit β7	Proteasomal protein degradation	0.36
Psme1	Proteasome activator	Proteasome function enhancement	0.35
Ufm1	Ubiquitin-like modifier	Protein turnover and ER stress responses	0.47
Nedd8	Ubiquitin-like protein	Cullin activation, ubiquitin ligase regulation	0.30
Atp6v1b2	V-ATPase subunit B2	Lysosomal acidification, autophagic degradation	0.45
Atp6v1g1	V-ATPase subunit G1	Lysosomal acidification	0.43
Lonp1	Mitochondrial protease	Mitochondrial protein quality control	0.40
Atad3	Mitochondrial AAA protein	Mitochondrial dynamics, mitophagy regulation	0.44
Park7 (DJ-1)	Stress response protein	Oxidative stress, aggrephagy support	0.36
Dync1i2	Dynein intermediate chain	Microtubule-based vesicle transport	0.35
Tubb4b	β-tubulin	Cytoskeletal trafficking, autophagosome movement	0.48
Ckap4	ER-associated protein	ER structure and protein trafficking	0.48

**Table 2:** Key proteins up- or downregulated in the *Gfpt1*-deficient C2C12 myoblast secretome compared with scramble. Proteins detected with an adjusted *p*-value of <0.05 were deemed significantly altered from scrambled control. Proteins with an adjusted *p*-value of <0.05 were deemed significantly altered from the control.

Protein	Description	Relevant Role	Fold Change
Eno1 (α-enolase)	Glycolytic enzyme; also moonlights as plasminogen receptor when extracellular	Promotes ECM remodeling and cell migration via plasmin activation; supports myoblast differentiation and tissue remodeling	1.7
A2m (Alpha-2-macroglobulin)	Broad-spectrum protease inhibitor	Regulates extracellular proteolysis, cytokine binding, and growth factor availability → impacts myogenesis and muscle repair environment	1.49
ApoA1	Major HDL apolipoprotein	Lipid transport and anti-inflammatory signaling support metabolic homeostasis in muscle and may influence differentiation under stress	1.45
Actb/Actg1 (β- and γ-actin)	Cytoskeletal proteins	Released during stress/damage; function as DAMPs (damage-associated signals); linked to cell motility and myoblast fusion dynamics	1.44
Fbln1 (Fibulin-1)	ECM glycoprotein	Regulates ECM organization, cell adhesion, and migration; important for myoblast alignment and differentiation niche	1.22
Anxa2 (Annexin A2)	Calcium-dependent phospholipid-binding protein	Membrane repair, vesicle trafficking, exosome release, and plasmin generation → key for myoblast membrane remodeling and fusion	0.67
Hspa5 (BiP/GRP78)	ER chaperone protein	ER stress marker; when secreted, acts as stress signal; indicates UPR activation and secretory pathway dysfunction in myoblasts	0.63
Histones (H2B variants)	Core nucleosome proteins	Extracellular histones act as alarmins → trigger inflammation, immune signaling, and cell stress responses; may reflect cell damage/death or vesicular release	0.35
Histones (H4)	Core nucleosome protein	Same as above; linked to inflammatory signaling, cytotoxicity, and stress-induced secretion	0.30
Histones (H2A variants)	Core chromatin proteins	Extracellular roles include immune modulation and DAMP signaling, indicating nuclear stress or impaired degradation	0.27
Histones (H3 variants)	Core chromatin proteins	Released under stress; may influence inflammatory and regenerative signaling in muscle environment	0.27
Hspa9 (Mortalin/GRP75)	Mitochondrial chaperone	Indicates mitochondrial stress/damage; involved in mitochondrial protein folding and quality control, relevant to mitophagy defects	0.09
Hspd1 (HSP60)	Mitochondrial chaperonin	Mitochondrial stress marker; extracellularly promotes immune signaling and stress adaptation; linked to metabolic dysfunction in myoblasts	0.01

## Data Availability

Data are contained within the article and Supplementary Materials. The mass spectrometry proteomics data have been deposited to the ProteomeXChange Consortium (ProteomeCentral Data and Tools) via the PRIDE partner repository with the data set identifier PXD079191.

## Supplementary Materials

The supporting information can be downloaded at: https://www.mdpi.com/article/10.3390/biom16070966/s1. Supplementary Figure S1: Supplementary experiments for Xbp1 expression determination; Supplementary Figure S2: p62 protein levels express a punctate pattern within Gfpt1-deficient myoblasts; Supplementary Figure S3: Srgn is retained by Gfpt1-deficient myoblasts, and has reduced protein secretion; Supplementary Table S1: RT-qPCR Primer Design. Supplementary File S1: Original images for Western Blot image.
