# Supplementary material for: Hexosamine Pathway Disruption by GFPT1 Loss Drives Coordinated Defects in Glycosylation, Autophagy, and Trafficking
Source: Biomolecules. 2026 Jun 30;16(7):966. doi: 10.3390/biom16070966 (PMC13406536; doi:10.3390/biom16070966)
Supplement: Supplementary file 1 [file biomolecules-16-00966-s001.zip › biomolecules-4323438-supplementary figures and table.pdf]

Hexosamine Pathway Disruption by GFPT1 Loss Drives Coordinated Defects in Glycosylation, Autophagy, and Trafficking

Stephen H. Holland [1,2,3], Ricardo Carmona-Martinez [1], Andreas Hentschel [4], Alexa Derksen [1,2], Kaela O'Connor [1,2], Daniel O'Neil [1], Kelly Ho [1,2], Stephen D. Baird [1], Andreas Roos [1,5,6], Sally Spendiff [1] and Hanns Lochmüller [1,2,3,7,8,9*]

1 Children's Hospital of Eastern Ontario Research Institute, Ottawa, ON, Canada. K1H8L1
sholl051@uottawa.ca (S.H.H.); rcarmona@cheo.on.ca (R.C.-M.); aderk098@uottawa.ca (A.D.); kocon090@uottawa.ca (K.O.); doneil@cheo.on.ca (D.O.); kho032@uottawa.ca (K.H.);
sbaird@arc.cheo.ca (S.B.); andreas.roos@uk-essen.de (A.R.); sspendiff@cheo.on.ca (S.S.)

2 Department of Cellular and Molecular Medicine, Faculty of Medicine, University of Ottawa,
Ottawa, ON, Canada, K1H 8M5.

3 Dr. Eric Poulin Center for Neuromuscular Disorders, Brain and Mind Research Institute, University of Ottawa, Ottawa, ON, Canada, K1H 8M5.

4 Leibniz-Institute for Analytical Sciences – ISAS- e.V, Dortmund, Germany, 44139.

5 Department of Pediatric Neurology, Centre for Neuromuscular Disorders in Children, University Duisburg-Essen, Hufelandstrasse 55, 45122 Essen, Germany

6 Department of Neurology with Heimer Institute for Muscle Research, University Hospital Bergmannsheil, Bochum, Germany, 44789.

7 Division of Neurology, Department of Medicine, The Ottawa Hospital, Ottawa, ON, Canada, K1H8L6.

8 Department of Neuropediatric and Muscle Disorders, Medical Center, University of Freiburg, Faculty of Medicine, Freiburg, Germany, 79106.

9 Centro Nacional de Analisis Genomico (CNAG-CRG), Center for Genomic Regulation, Barcelona Institute of Science and Technology (BIST), Barcelona, Spain, 08028.

* Correspondence: hlochmuller@toh.ca

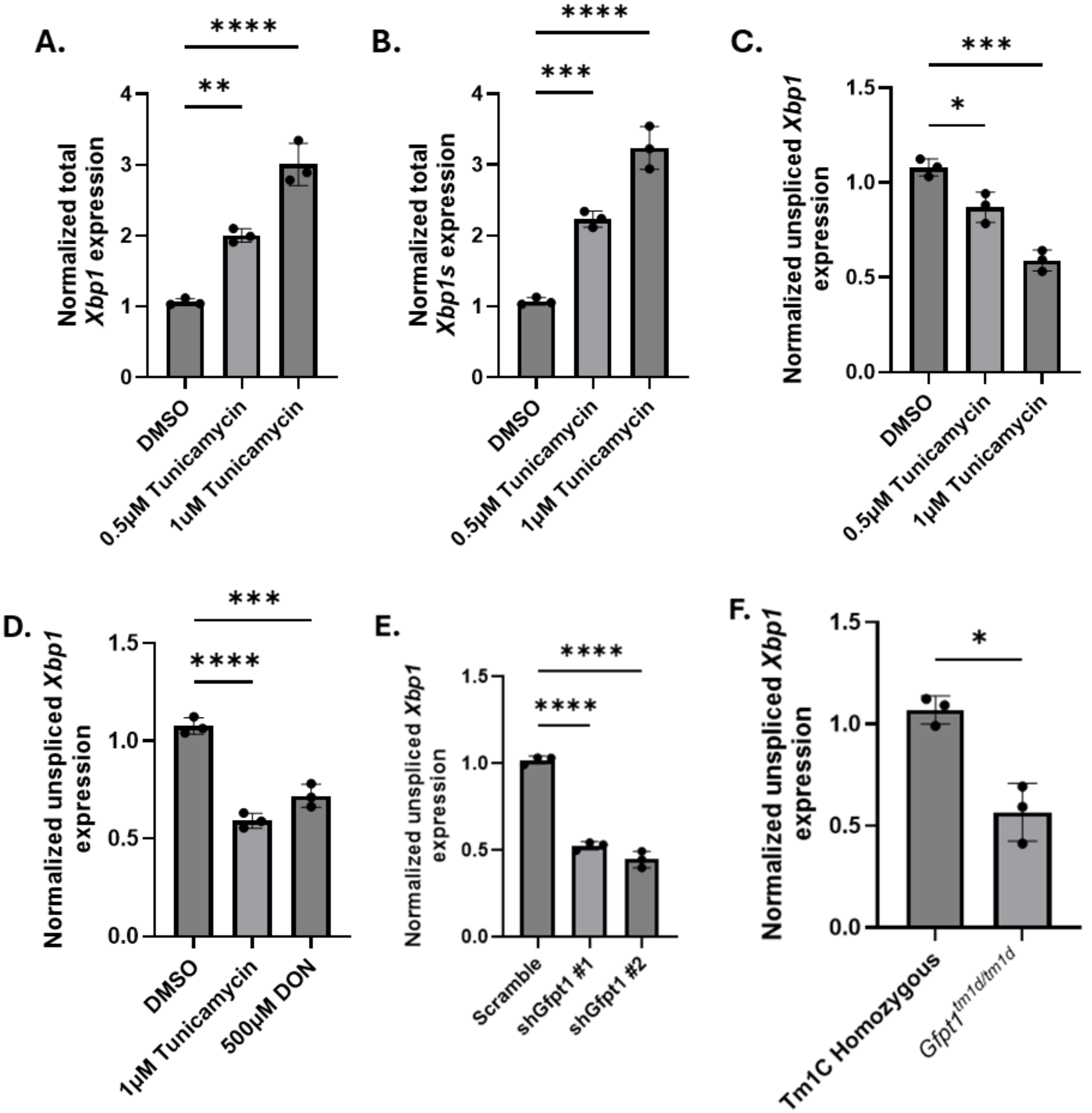

Supplementary Figure S1: Supplementary experiments for *Xbp1* expression determination: A. Dose–response analysis of tunicamycin, an N-linked glycosylation inhibitor that induces glycosylation stress. RT-qPCR revealed a dose-dependent increase in total Xbp1 expression**. B.** Increasing doses of tunicamycin led to elevated expression of spliced *Xbp1s*, and **C.** a corresponding decrease in unspliced *Xbp1u***. D.** Treatment with tunicamycin or DON significantly reduced unspliced *Xbp1u* expression compared to untreated controls. **E.** Gfpt1-deficient C2C12 myoblasts treated with doxycycline (shGfpt1 #1 and shGfpt1 #2) exhibited a significant decrease in unspliced *Xbp1u* expression by RT-qPCR. **F.** RT-qPCR performed on quadriceps muscle from *Gfpt1*$^{tm1d/tm1d}$ mice and *Tm1c* control mice showed reduced unspliced *Xbp1u* expression in *Gfpt1*$^{tm1d/tm1d}$

tissue. Three biological replicates were analyzed for each experiment. Statistical significance was determined by one-way ANOVA for panels B–E, and by Student's *t*-test for F–G. Gene expression was normalized to the geometric mean of Rpl27, Gapdh, and Actb. * $p < 0.05$; ** $p < 0.01$; *** $p < 0.001$; **** $p < 0.0001$.

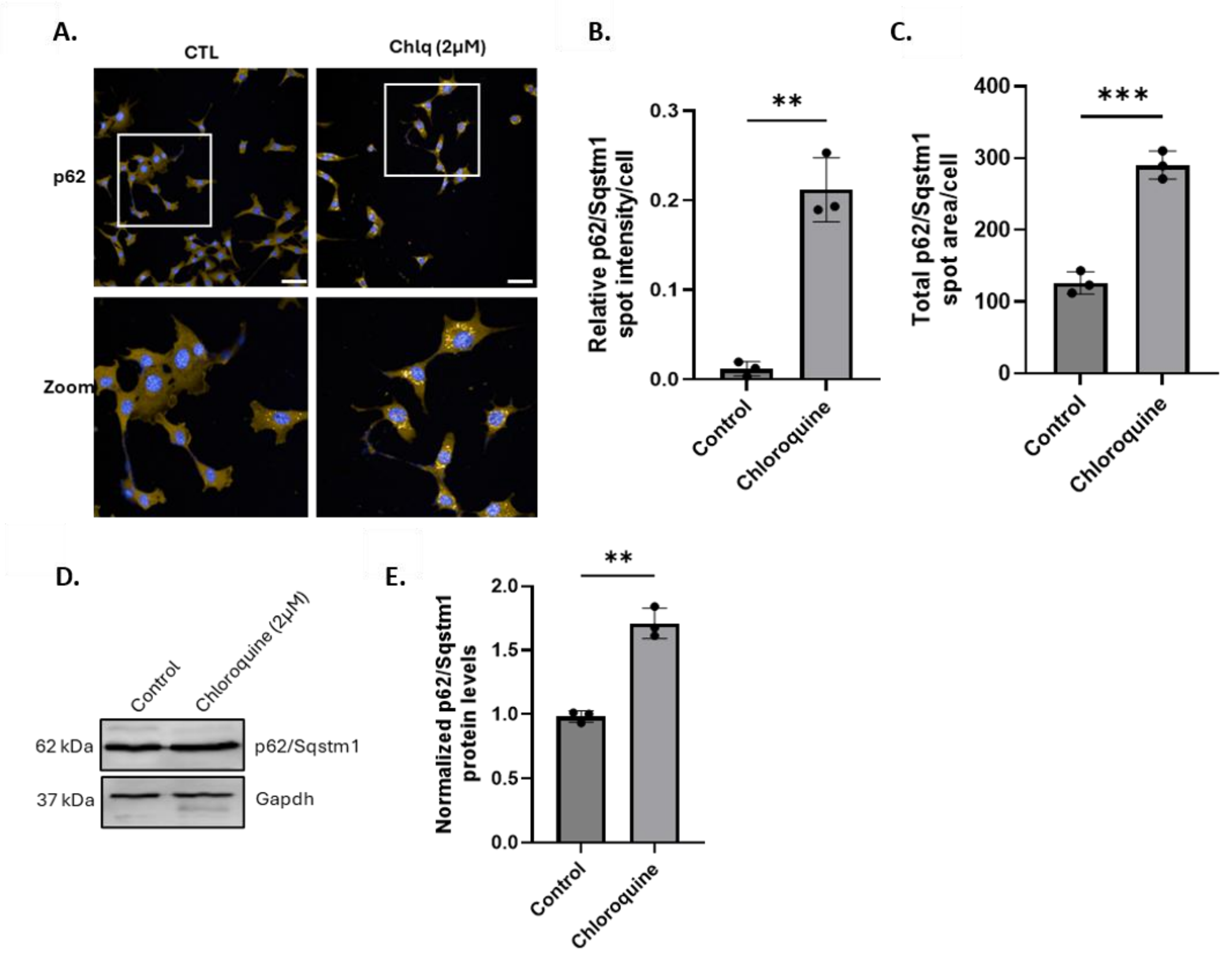

Supplementary Figure S2: p62 protein levels express a punctate pattern within Gfpt1-deficient myoblasts. A. Immunofluorescence staining of C2C12 myoblasts treated with 25 µM chloroquine and labeled for p62/SQSTM1. Images were acquired at 40× magnification using the OPERA™ high-content imaging system. Scale bar = 10 µm. **B.** Image analysis using Columbus™ software shows that chloroquine treatment increases p62/SQSTM1 staining intensity, **C.** increases the average number of p62-positive puncta per cell compared with untreated controls. **D.** Western blot analysis of soluble and insoluble fractions from control and Gfpt1-deficient C2C12 myoblasts demonstrates accumulation of p62/SQSTM1 in both fractions, with or without chloroquine treatment. **E.** Quantification shows elevated levels of soluble p62/SQSTM1 in Gfpt1-deficient myoblasts. All graphs show mean ± SD. Statistical significance was determined using one-way ANOVA. All experiments were performed with three biological replicates per group. Comparisons between control and Gfpt1-deficient samples: ** $p < 0.01$, *** $p < 0.001$.

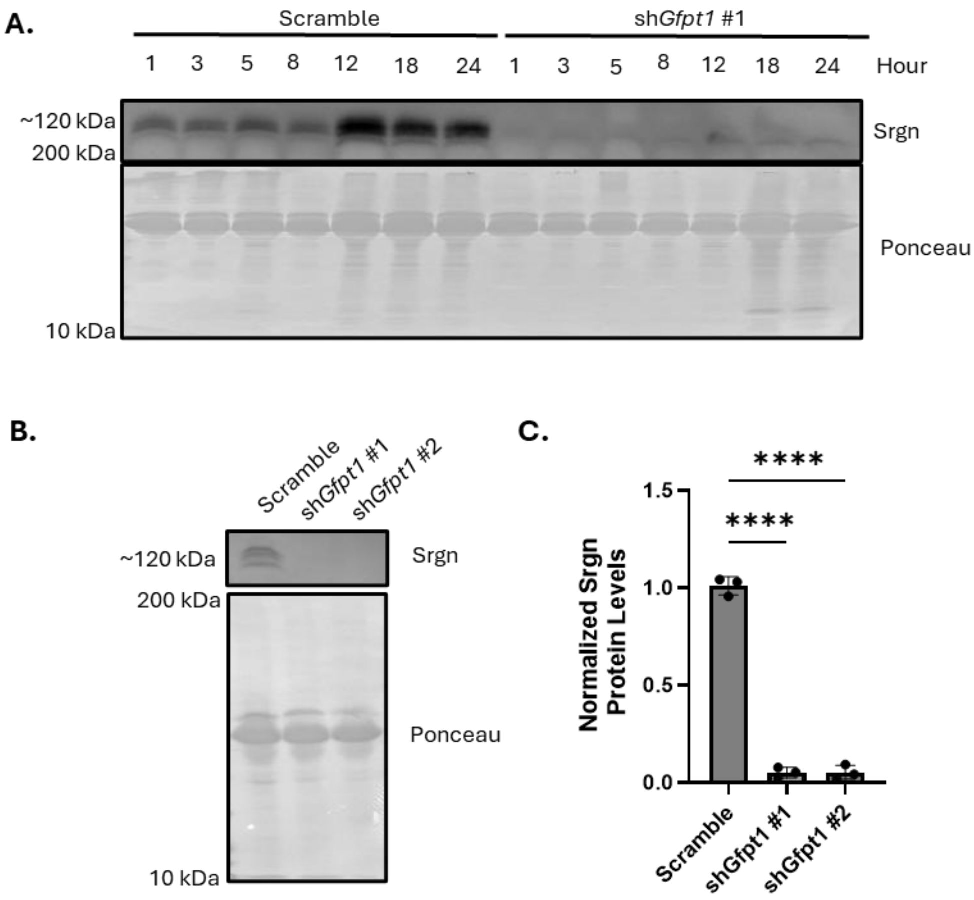

Supplementary Figure S3: Srgn is retained by Gfpt1-deficient myoblasts, and has reduced protein secretion. A. Western blot analysis of conditioned medium collected over a 24-hour time course from control and shGfpt1 #2 C2C12 myoblasts, alongside SRGN protein levels in the insoluble fraction of Gfpt1-deficient myoblasts. **B.** Western blot of conditioned medium collected at 24 hours from control and Gfpt1-deficient C2C12 myoblasts showing reduced secretion of SRGN. **C.** Quantification demonstrates a significant reduction in high–molecular-weight SRGN species in Gfpt1-deficient myoblasts. All graphs show mean ± SD. Statistical significance was determined using one-way ANOVA. All experiments were performed with three biological replicates per group. Comparisons between control and Gfpt1-deficient samples: **** $p < 0.0001$.

Supplementary Table S1: RT-qPCR Primer Design.

Target Gene	Forward Primer	Reverse Primer
Unspliced Xbp1	5′-cagactacgtgcacctctgc-3′	5′-cagggtccaacttgaccagaat-3′
Spliced Xbp1	5′-gctgagtccgcagcaggt-3′	5′-cagggtccaacttgaccagaat-3′
Total Xbp1	5′-tgaaaaacagagtagcagcctaga-3′	5′cccaagcgctttcttcactc-3′
Gapdh	5′- ctcccactcttccaccttcg-3′	5′- gcctctcttgctcagtgtcc-3′
Ppia	5′- gccttcctcctttcacagaa-3′	5′- gatgccaggacctgtatgct-3′
Rpl27	5′- aagccgtcatcgagaaca-3′	5′- cttgatcttggatcgcttggc-3′
Gfpt1	5′- ccaacgcctgcaaaatccag-3′	5′-ttctccatgtgtcgcccaac-3′
