# Supplementary figures and images for: Hexosamine Pathway Disruption by GFPT1 Loss Drives Coordinated Defects in Glycosylation, Autophagy, and Trafficking
Source: Biomolecules. 2026 Jun 30;16(7):966. doi: 10.3390/biom16070966 (PMC13406536; doi:10.3390/biom16070966)

Figure 1B

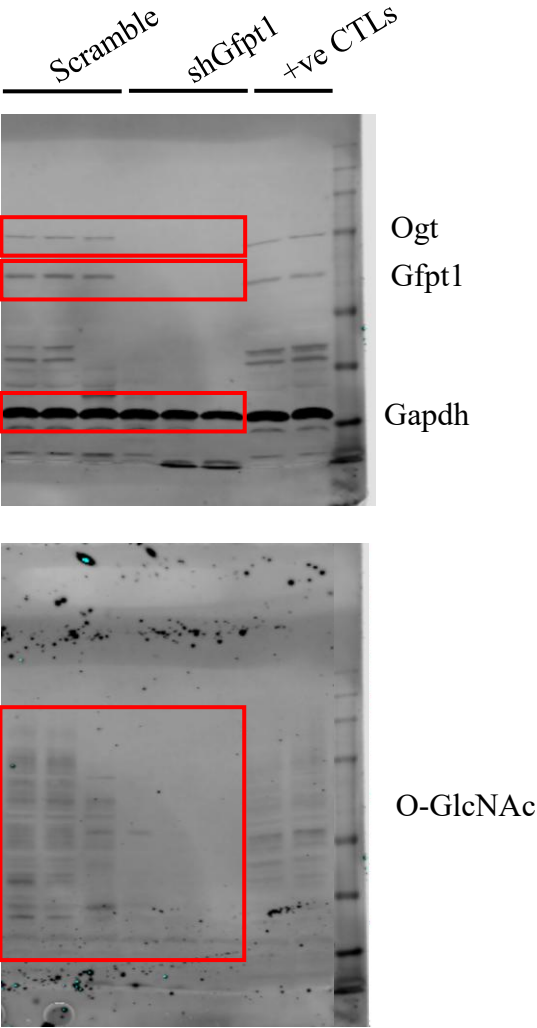

Figure 3A + Figure S2D

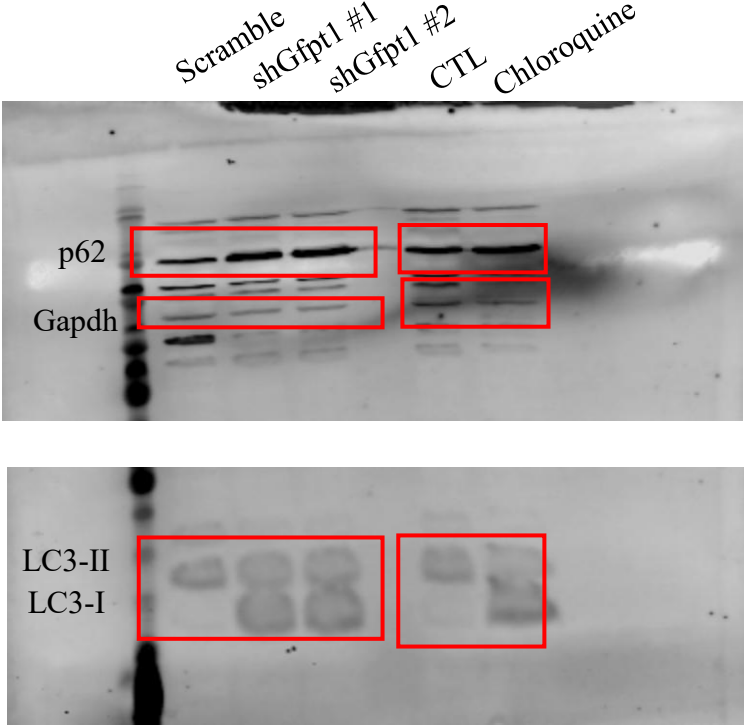

Figure 4A

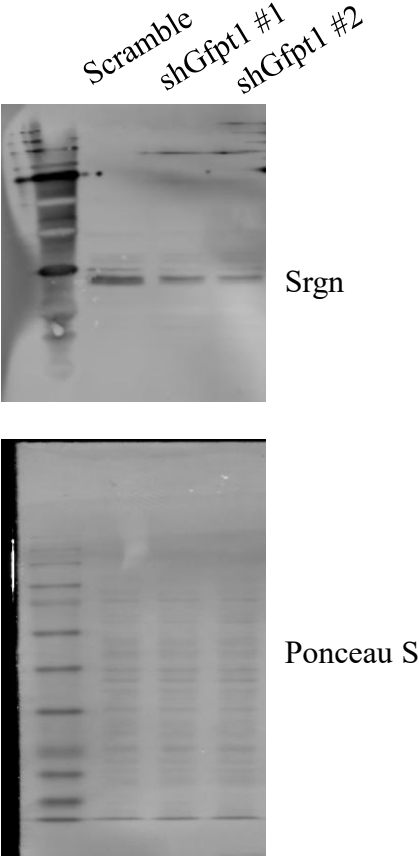

Figure 4C

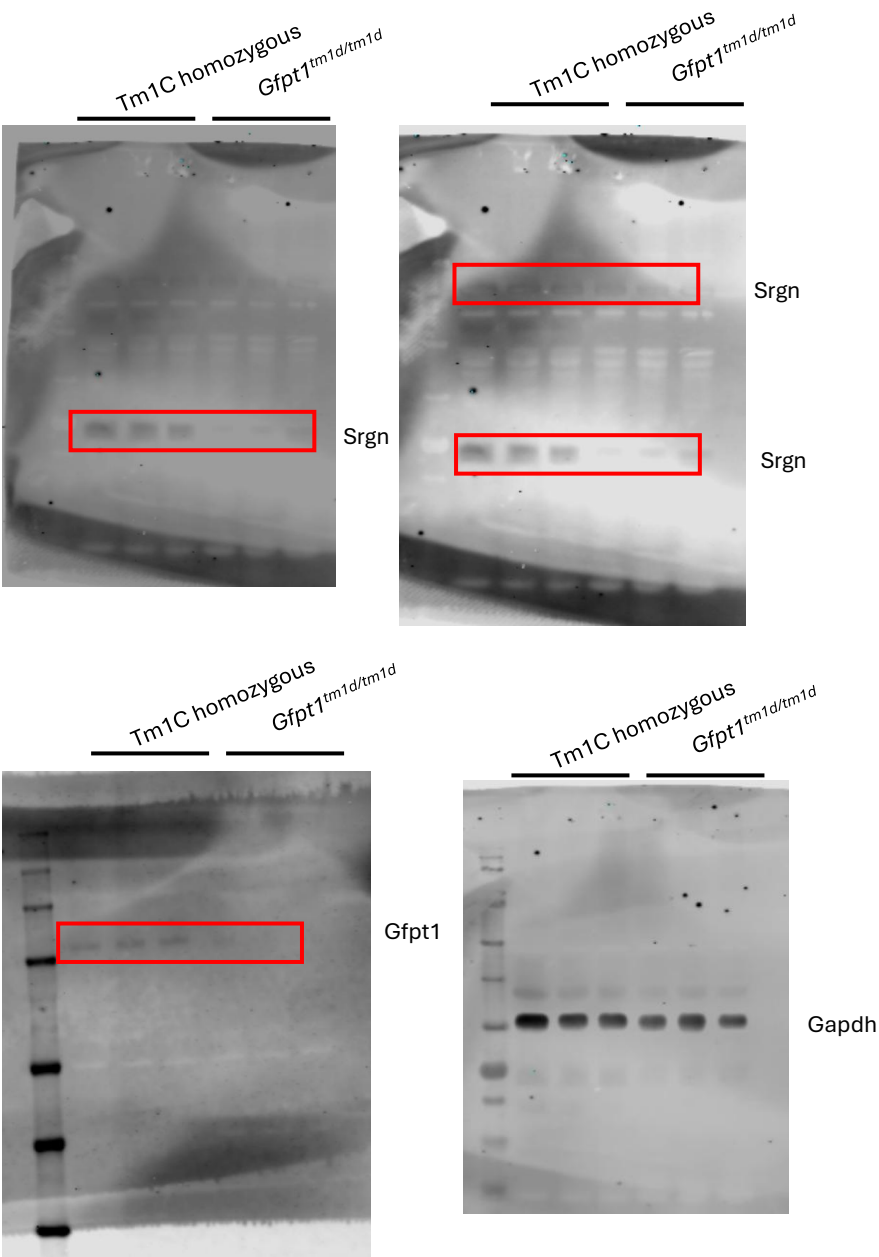

Figure 4I

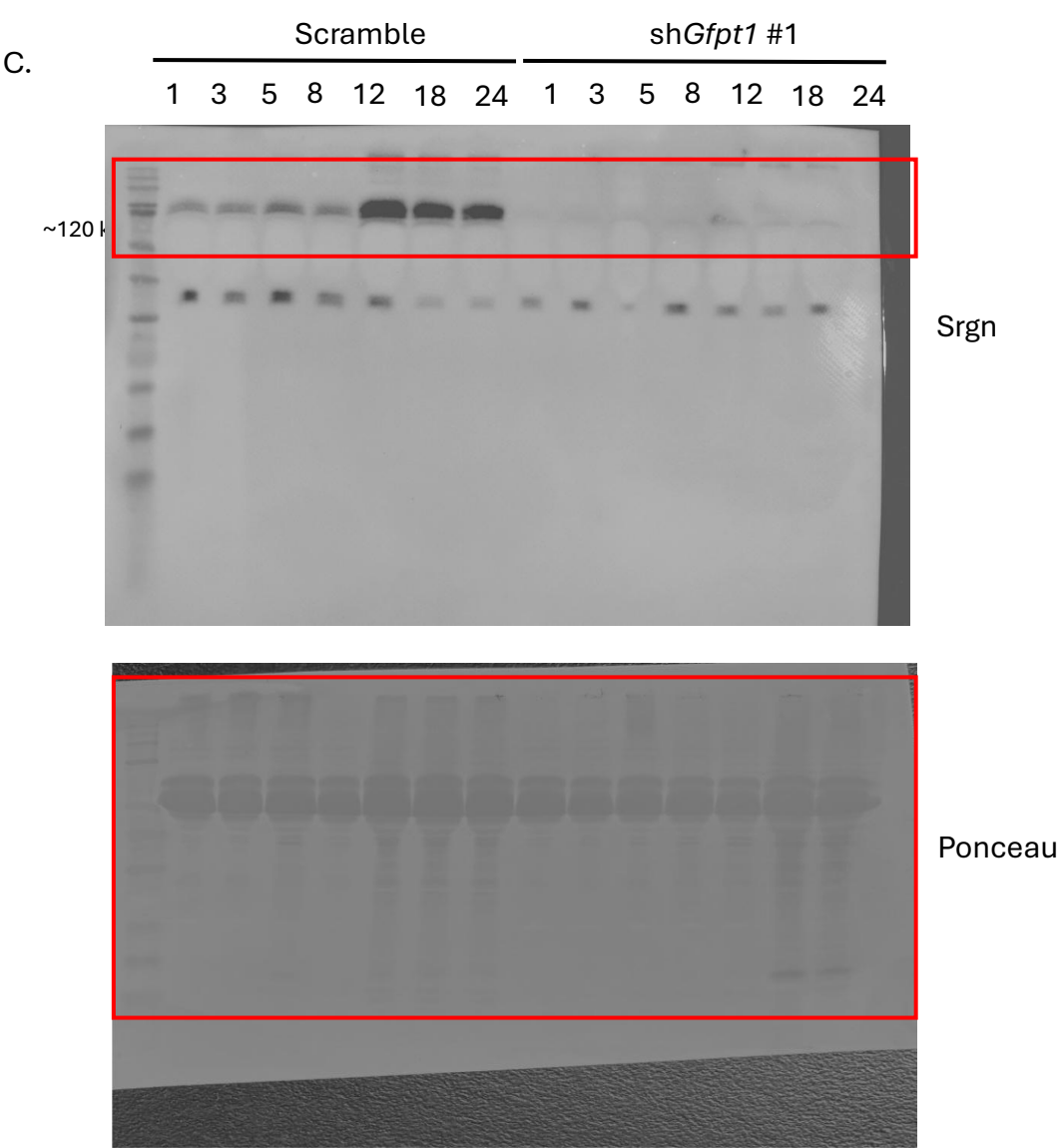

Figure S3A

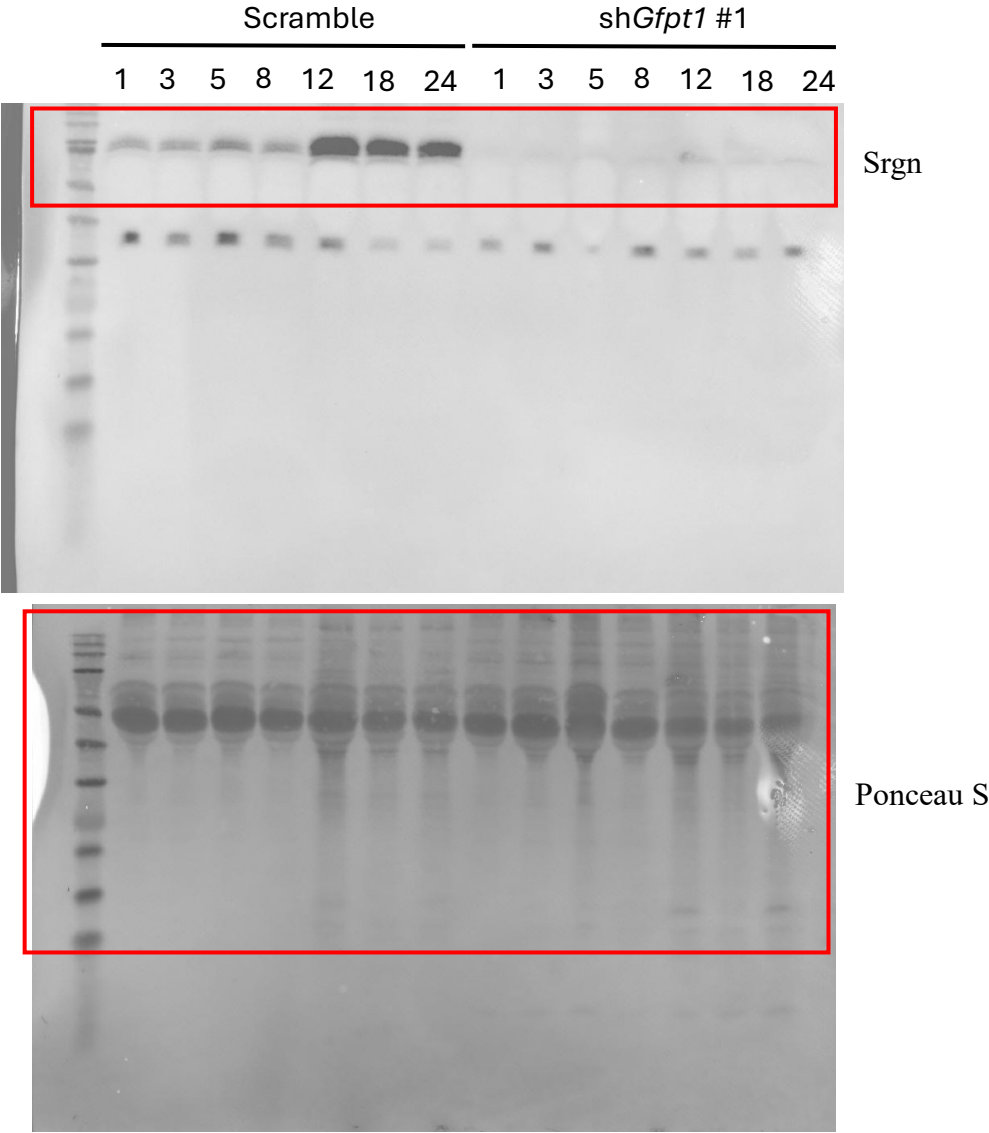

Figure S3B

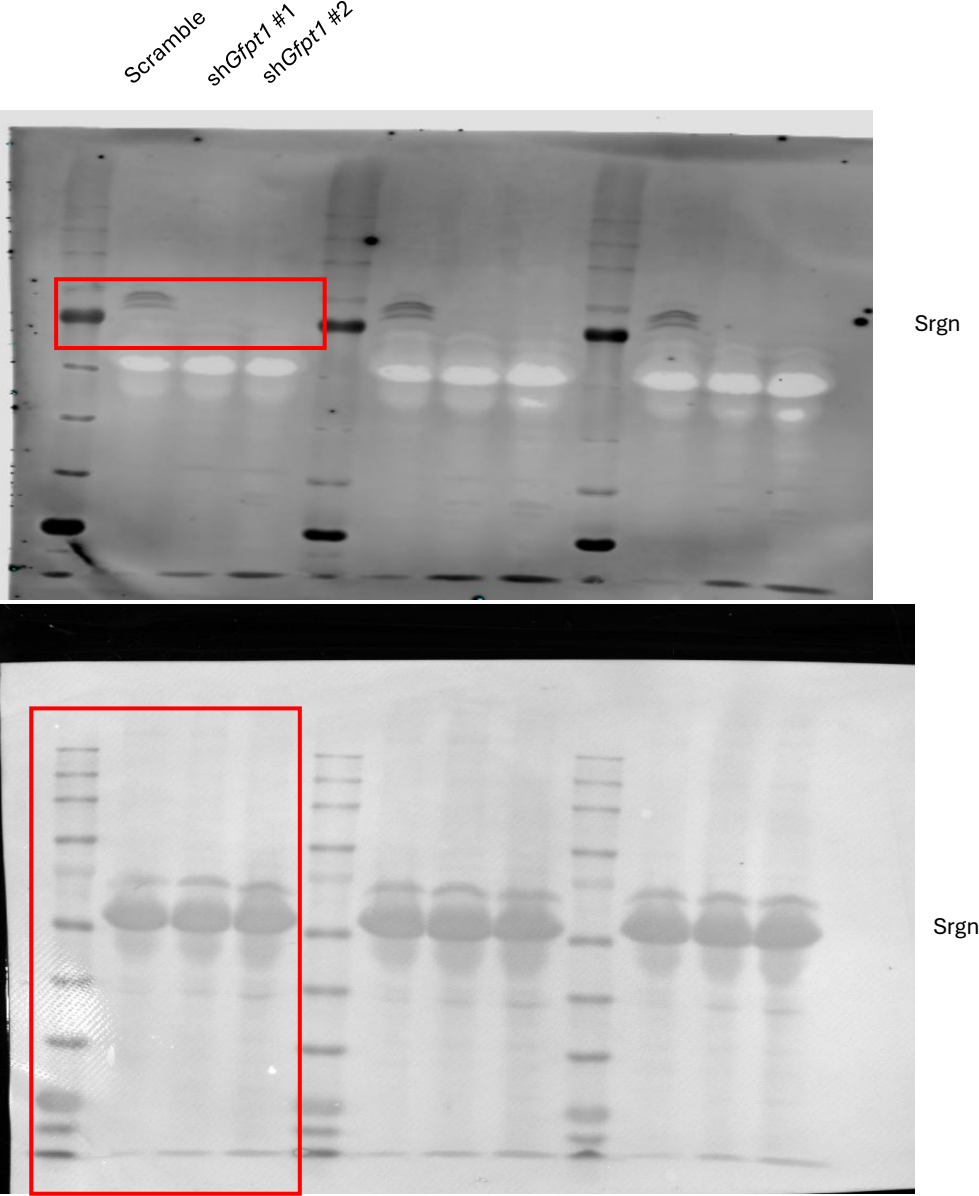

Supplement: Supplementary file 1 [file biomolecules-16-00966-s001.zip › biomolecules-4323438-Supplementary File S1 Original images for Western Blot image.pdf.pdf]
